# Diagnosis and management of acute appendicitis. EAES consensus development conference 2015

**DOI:** 10.1007/s00464-016-5245-7

**Published:** 2016-09-22

**Authors:** Ramon R. Gorter, Hasan H. Eker, Marguerite A. W. Gorter-Stam, Gabor S. A. Abis, Amish Acharya, Marjolein Ankersmit, Stavros A. Antoniou, Simone Arolfo, Benjamin Babic, Luigi Boni, Marlieke Bruntink, Dieuwertje A. van Dam, Barbara Defoort, Charlotte L. Deijen, F. Borja DeLacy, Peter MNYH Go, Annelieke M. K. Harmsen, Rick S. van den Helder, Florin Iordache, Johannes C. F. Ket, Filip E. Muysoms, M. Mahir Ozmen, Michail Papoulas, Michael Rhodes, Jennifer Straatman, Mark Tenhagen, Victor Turrado, Andras Vereczkei, Ramon Vilallonga, Jort D. Deelder, Jaap Bonjer

**Affiliations:** 1 Department of Surgery, VU University Medical Centre, Amsterdam, The Netherlands; 2 Department of Surgery, Red Cross Hospital, Beverwijk, The Netherlands; 3 Department of Surgery, Spaarne Gasthuis, Haarlem, The Netherlands; 4 Department of Surgery, St Mary’s Hospital, London, UK; 5 Department of Surgery, Center for Minimally Invasive Surgery, Neuwerk Hospital, Mönchengladbach, Germany; 6 Department of Surgery, University Hospital of Heraklion, Heraklion, Greece; 7 Department of Surgery, University of Torino, Torino, Italy; 8 Department of Surgery, Agaplesion Markus Krankenhaus, Frankfurt am Main, Germany; 9 Department of Surgery, Minimally Invasive Surgery Research Center, University of Insubria, Varese, Italy; 10 Department of Surgery, Maria Middelares Ghent, Ghent, Belgium; 11 Department of Surgery, Hospital Clinic of Barcelona, Barcelona, Spain; 12 Department of Surgery, St. Antonius Hospital, Nieuwegein, The Netherlands; 13 Department of Surgery, Noordwest Clinics Alkmaar, Alkmaar, The Netherlands; 14 Department of Surgery, University of Medicine and Pharmacy “Carol Davila”, Bucharest, Romania; 15 Medical Library, Vrije Universiteit, Amsterdam, The Netherlands; 16 Department of Surgery, School of Medicine, Bahcesehir University, Istanbul, Turkey; 17 Department of Surgery, Tel Aviv Sourasky Medical Centre, Tel Aviv, Israel; 18 Department of Surgery, Stepping Hill Hospital, Stockport, UK; 19 Department of Surgery, Hospital de la Santa Creu i Sant Pau, Barcelona, Spain; 20 Department of Surgery, Medical School University of Pécs, Pecs, Hungary; 21 Department of Surgery, University Hospital Vall Hebrón, Barcelona, Spain; 22 Department of Pediatric Surgery, VU University Medical Centre, P.O. Box 22660, 1100 DD Amsterdam, The Netherlands

**Keywords:** Appendicitis, Uncomplicated appendicitis, Complicated appendicitis, Appendectomy, Laparoscopic appendectomy

## Abstract

Unequivocal international guidelines regarding the diagnosis and management of patients with acute appendicitis are lacking. The aim of the consensus meeting 2015 of the EAES was to generate a European guideline based on best available evidence and expert opinions of a panel of EAES members. After a systematic review of the literature by an international group of surgical research fellows, an expert panel with extensive clinical experience in the management of appendicitis discussed statements and recommendations. Statements and recommendations with more than 70 % agreement by the experts were selected for a web survey and the consensus meeting of the EAES in Bucharest in June 2015. EAES members and attendees at the EAES meeting in Bucharest could vote on these statements and recommendations. In the case of more than 70 % agreement, the statement or recommendation was defined as supported by the scientific community. Results from both the web survey and the consensus meeting in Bucharest are presented as percentages. In total, 46 statements and recommendations were selected for the web survey and consensus meeting. More than 232 members and attendees voted on them. In 41 of 46 statements and recommendations, more than 70 % agreement was reached. All 46 statements and recommendations are presented in this paper. They comprise topics regarding the diagnostic work-up, treatment indications, procedural aspects and post-operative care. The consensus meeting produced 46 statements and recommendations on the diagnostic work-up and management of appendicitis. The majority of the EAES members supported these statements. These consensus proceedings provide additional guidance to surgeons and surgical residents providing care to patients with appendicitis.

**Electronic supplementary material:**

The online version of this article (doi:10.1007/s00464-016-5245-7) contains supplementary material, which is available to authorized users.

Acute appendicitis is a common gastrointestinal disease affecting 5.7–57/per 100.000 individuals each year with the highest incidence in children and adolescents [1–6]. The variation of incidence is due to variations in ethnicity, sex, age, obesity and season of the year [3, 6–11]. Based upon the entrenched idea that appendicitis is an irreversible progressive disease eventually leading to perforation, removal of the appendix is the gold standard of treatment. The medical profession has gained much experience in managing patients with acute appendicitis ever since Fitz’s first report in 1886 [12]. Large heterogeneity exists, however, between existing intercontinental, European and national guidelines regarding diagnosing and managing acute appendicitis. For instance, in the Netherlands, pre-operative imaging studies are promoted and considered mandatory in order to prevent negative appendectomies according to national guidelines, whereas in guidelines of other countries, it is not promoted nor considered mandatory [13]. Another example is the inconsistency regarding the management of an unexpected “normal appendix” during diagnostic laparoscopy [13, 14]. This heterogeneity prompted the need for an European consensus development conference for the diagnosis and management of acute appendicitis.

The European Association of Endoscopic Surgery (EAES) initiated a consensus development conference meeting on the management of acute appendicitis for its 2015 meeting in Bucharest. The aim of this consensus meeting was to develop practical guidelines based on the available evidence combined with the expertise of a selected panel of EAES surgeons. The findings are reported in this manuscript.

## Materials and methods

The coordinating team (HJB, RG, HE and MGS) invited ten surgeons from nine European countries to serve as experts in this consensus development conference. An international research team of 16 young surgical researchers across 11 European countries was formed to evaluate and process the existing literature on the management of acute appendicitis. The coordinators generated a list of topics on acute appendicitis to be addressed (Appendix 1). An exploratory literature search was conducted in order to identify any additional topics of interest. All topics were approved by the experts and subsequently divided into three main parts: pre-operative care, operative care and after care. Based upon the topics, research questions were formulated, reviewed and approved by the panel of experts.

### Literature search and processing of the literature

Research questions were used as guidance to conduct literature searches. The searches were conducted in cooperation with a medical information specialist of the Vrije Universiteit. Searches were performed in the following databases: PubMed, Web of science and the Cochrane library from inception up to 31 December 2014. No limitation was used regarding year of publication. Searches have been attached in Appendix 2. All papers published in European languages, and all study types with the exception of case reports were included in the search.

All articles were screened and reviewed by teams of two research fellows for eligibility, based on title and abstract. If eligible for inclusion, full text articles were obtained. If no full text was available, the article was excluded. In case of disagreement between the two research fellows, the coordinator dedicated to the topic acted as referee. Full text articles were summarized, evaluated and discussed at research meetings to assess their eligibility for inclusion in the review process. All included studies were evaluated according to the GRADE system [15–18]. The GRADE system systematically evaluates the available literature and focuses on the level of evidence based upon the types of studies included. The level of evidence can be marked as high, moderate, low or very low. This could be either downgraded in case of significant bias or upgraded when multiple high-quality studies showed consistent results. The highest levels of evidence (systematic reviews) were assessed first. If the systematic review was of sufficient quality, it was used to answer the research question. If no systematic review of sufficient quality was found, randomized controlled trials (RCTs) and cohort studies were evaluated. All selected studies were uploaded to a Mendeley database that was accessible to all research fellows, coordinators and experts.

After the literature search, an expert was assigned to every couple of researchers. This threesome was assigned research questions from the pre-operative care, operative care and after care. Hereafter they were responsible for formulating a statement/conclusion and, if possible, a recommendation on the assigned research questions. Again, the quality of the evidence was evaluated according to the GRADE/SIGN system [15–19]. The strength of the recommendation was based on the level of evidence and qualified as weak or strong. This was reflected in terms, using “*recommend”* in case of a strong recommendation and “*suggest”* in case of a weak recommendation.

A face-to-face consensus meeting among the experts was held in Amsterdam on the 1 May 2015 to discuss the final statements and recommendations. The coordinating team all experts and members of the international research team attended the meeting. A modified Delphi method was used. The Delphi method is a structured process, commonly used to develop healthcare quality indicator and consists of four key components; iteration, controlled acquisition of feedback, aggregation of responses and anonymity. As anonymity was not applicable in our situation, we used the term modified [20–22]. All statements and recommendations were shared with proposed levels of evidence with the entire group. After displaying the statements and recommendations, the experts casted their votes of agreement or disagreement. Refrain from voting was not allowed. No discussion was allowed between the experts at this point of time. In case of 100 % consensus, the statement and recommendation were accepted without further voting or discussion. In case of lack of consensus, the research team responsible for the statement presented the underlying considerations. After discussion between the experts, a second voting round was conducted. The statement or recommendation was accepted in case of at least 70 % consensus. Those statements and recommendations with less than 70 % consensus in the expert meeting were not included in the web survey or in the 2015 Bucharest meeting.

All finalized recommendations and statements with levels of evidence were entered into a web survey and distributed to all EAES members by e-mail. The web survey was open from 27 May until 3 July 2015. The recommendations or statements as well as the levels of evidence were open to several voting options: “agree”, “partly agree”, “disagree” or “don’t know”. The option “partly agree” meant that the voter agreed with the recommendation, but did not agree with the strength of recommendation.

All finalized recommendations and statements from the Amsterdam meeting with levels of evidence were presented at a plenary session of the 23rd annual meeting of the EAES on the 5 June 2015 in Bucharest. Live voting was performed using a digital voting system. Voting options were the same as the abovementioned.

Both results from the web survey and the Bucharest meeting are presented in the “Results” section.

## Results

**Fig. 1:**
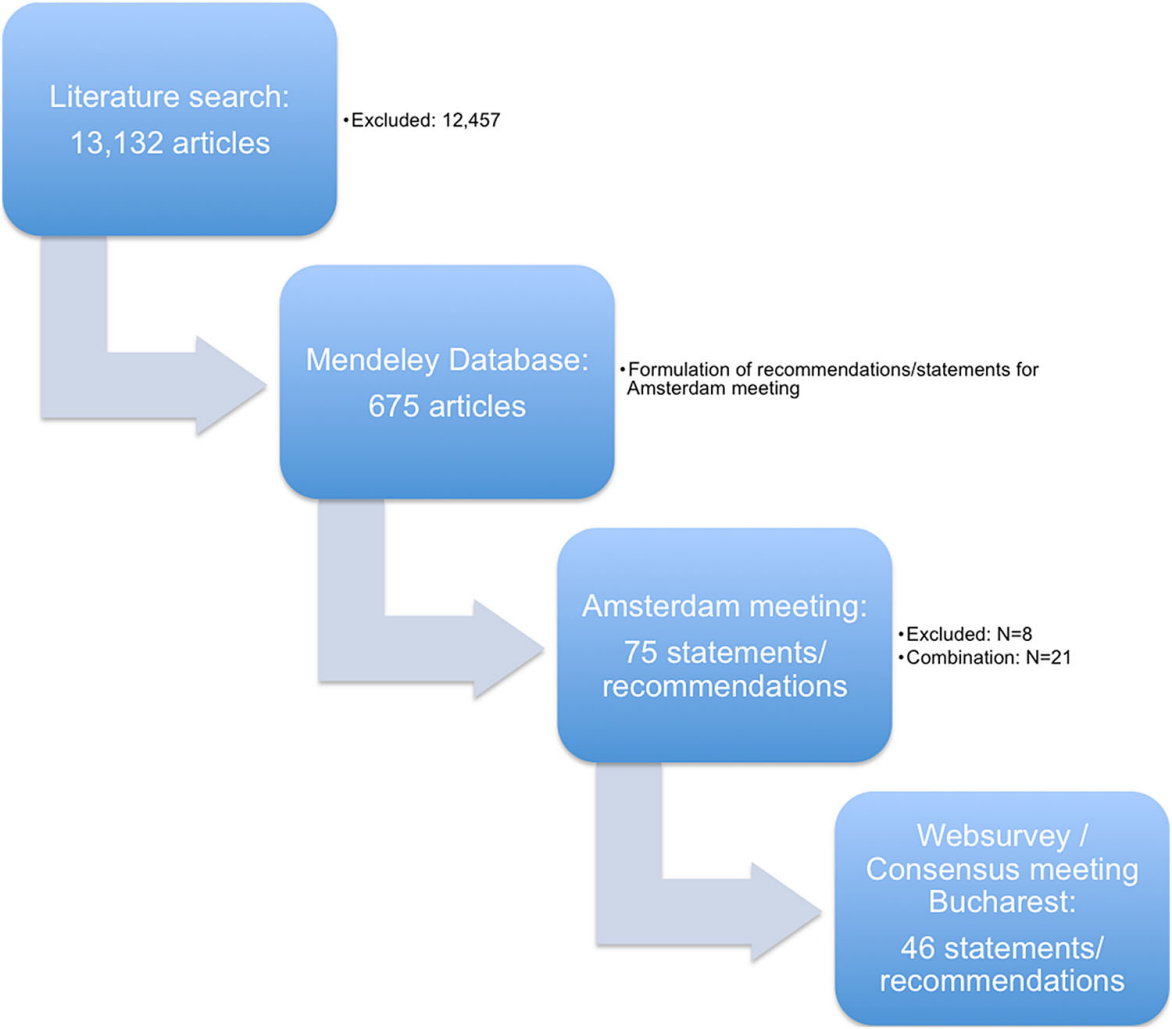
Flow diagram of the process prior to the EAES consensus meeting in Bucharest 2015

The literature search yielded 13,132 articles. The title, abstract and full text were reviewed. In total, 675 articles were selected and reviewed in detail to define 75 statements and recommendations, which were subsequently discussed at the Amsterdam meeting. (Appendix 1) During this meeting, the following statements and recommendations were excluded: on incidence and prevalence of appendicitis (*n* = 4), on the place of NOTES in acute appendicitis (*n* = 1), on the learning curve of appendectomy (*n* = 1), on day surgery for acute appendicitis (*n* = 1) and on the skeletonizing technique of the meso-appendix (*n* = 1). Twenty-one statements were combined leaving a total of 46 statement and recommendations; 8 statements and 14 recommendations for pre-operative care, 1 statement and 15 recommendations for operative care and 2 statements and 6 recommendations for aftercare (Fig. 1). Of the 675 articles, 100 were excluded due to the fact that statements and recommendations were excluded or were combined, rendering 575 articles (Fig. 1; Appendix 3).

### Web survey

In total, 317 EAES members responded to the web survey; 90 % were surgeons and 10 % surgical residents.

### Bucharest meeting

The 2015 EAES congress in Bucharest was attended by 1166 delegates. During the plenary consensus meeting, 232 delegates voted. Sixty-eight per cent were surgeons, 26 % surgical residents and 6 % scientists, physician assistants and others.

## Pre-operative care

Establishing the diagnosis of acute appendicitis remains challenging. The clinical presentation of acute appendicitis can vary from mild symptoms to signs of generalized peritonitis and sepsis. Hence, the value of individual clinical variables to determine the likelihood of acute appendicitis in a patient is low [23, 24]. Biochemical testing is performed routinely in most patients. Its value in confirming acute appendicitis is debatable. A recent systematic review showed that elevated C-reactive protein levels render the highest diagnostic accuracy followed by increased numbers of leucocytes with an area under the curve of 0.75 [95 % CI 0.71–0.78] and 0.72 [95 % CI 0.68–0.76], respectively [24]. The area under the curve represents the ability of a test to correctly classify patients. In case the score is between the 0.7 and 0.8, it represents a fair test. Both clinical and biochemical variables have been combined into clinical predicting rules (CPR) such as the Alvarado score and paediatric appendicitis score (PAS) [25, 26]. This was done to increase the value of the individual variables. Ohle et al. [27] demonstrated that the Alvarado score was good at “ruling-out” appendicitis with an overall sensitivity and specificity of 96 and 81 %, respectively. In children, however, it has been shown that the PAS outperforms the Alvarado score [28]. To increase the predictive value of these two tests Ebell et al. [29] identified new cut-off values for the Alvarado score and PAS, which improved sensitivity and specificity rates. Based upon the Alvarado score, patients can now be categorised into low risk (Score < 4), intermediate (4–8) and high risk (≥9). The use of such CPRs appears useful to determine the likelihood of acute appendicitis. Distinguishing between low, intermediate and high risk provides guidance whether imaging studies are necessary.

Imaging studies in patients with a clinical suspicion of acute appendicitis can reduce the negative appendectomy rate, which has been reported to be as high as 15 %. Ultrasonography, abdominal computed tomography (CT) and magnetic resonance imaging (MRI) are most commonly used. Ultrasonography is non-invasive, avoids radiation and is associated with a sensitivity rate between 71 and 94 % and a specificity rate between 81 and 98 %. The positive likelihood ratio of ultrasonography is high at values between 6 and 46, while the negative likelihood ratio is moderate (0.08–0.30) [30–39]. Ultrasonography is therefore reliable to confirm presence of appendicitis but unreliable to exclude appendicitis. Furthermore, one should bear in mind that ultrasonography is highly operator dependent. Inconclusive ultrasonography findings, mostly due to failure visualizing the appendix, mandate additional imaging studies.

Abdominal computed tomography (CT) for suspected appendicitis has sensitivity and specificity rates between 76–100 % and 83–100 %, respectively, and, therefore, is superior to ultrasonography. Lower values of sensitivity and specificity can be explained by the use of enteral contrast [32, 33, 35–44]. However, the radiation exposure of abdominal CT is a concern particularly in children and during pregnancy. The estimated lifetime cancer-related mortality risk of developing a radiation-induced malignancy is approximately 0.18 % for a 1-year-old child and 0.11 % in a 15-year-old child if an abdominal CT is performed [45, 46]. Computed tomographies employing only a quarter of the standard radiation dose (low-dose CTs) provide similar imaging results as standard CTs and are, hence, an excellent alternative [47]. Regarding the administration of oral contrast, Andersson et al. [48] concluded in their meta-analysis that a CT scan without oral contrast was superior to CTs with oral contrast in terms of sensitivity and specificity. Therefore, low-dose CTs without oral contrast are preferable in patients with suspected appendicitis [48].

Magnetic resonance imaging (MRI) is used in pregnant patients and children with inconclusive findings at ultrasonography [49]. A recent meta-analysis on MRI in 363 patients with appendicitis, yielded a sensitivity rate of 97 % [95 % CI 92–99 %], a specificity rate of 95 % [95 % CI 94–99 %], a positive likelihood ratio of 16.3 [95 % CI 9.10–29.10] and a negative likelihood ratio of 0.09 [95 % CI 0.04–0.20] [50]. These rates are comparable to those of CT imaging, although these findings should be interpreted with care as most studies have been performed in a selected group of patients. MRI is associated with significant costs, and interpreting the images requires experience. Therefore, at the present time, use of MRI appears limited to pregnant women and children.

**Fig. 2:**
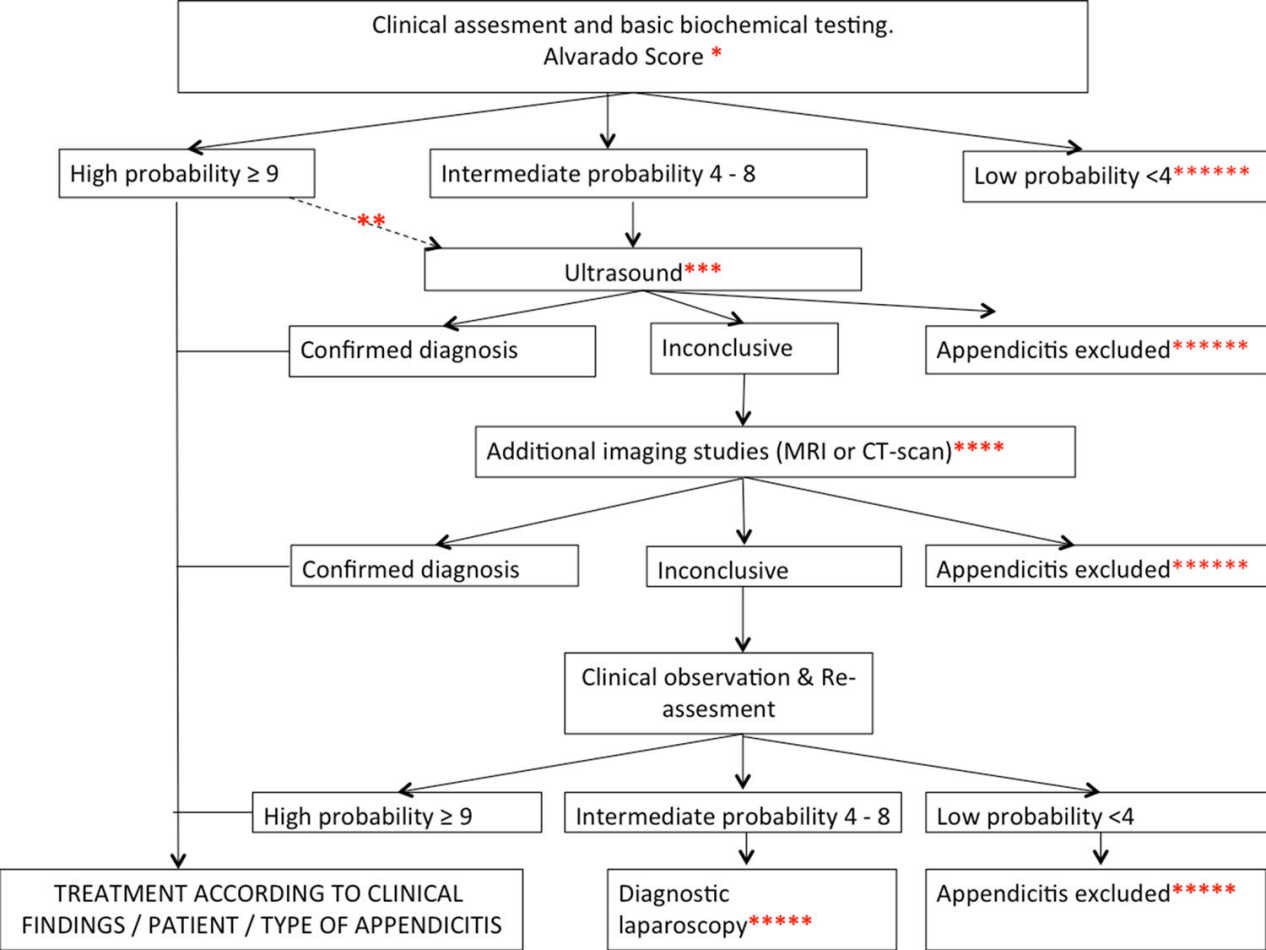
Algorithm. *The cut-off values are based upon the study by Ebell et al. [29]. **One could consider performing additional imaging studies in patients with high probability based upon the Alvarado score in order to reduce the negative appendectomy rate. ***Ultrasound should be performed as a first level diagnostic imaging study, although in specific patient groups (such as the obese) an immediate CT scan might be considered. ****In case of an inconclusive result from the ultrasound, we recommend that additional imaging studies should be performed. Either a CT or MRI is preferred although it is recommended to perform an MRI in children and pregnant patients. It is therefore obligated to rule out pregnancy before a CT scan is obtained in a woman of reproductive age suspected of appendicitis. *****In case all the imaging studies are inconclusive, patients should be observed and reassessed. Diagnostic laparoscopy should be reserved for those patients with a continuous high index of suspicion after reassessment. ******In case of low probability based upon the Alvarado score, other diagnoses should be excluded and the patient can be either discharged with good instruction (with an optional reassessment the next day) or admitted for observation if the clinical condition mandates this. In case appendicitis is excluded, patients should be treated for the set diagnosis according to the local protocols

The algorithm associated with the Alvarado score (recommendation 4) is shown in Fig. 2.

In obese patients (definition depends on the reference study), the diagnostic accuracy of ultrasound is diminished due to an increase of the subcutaneous and intra-abdominal fat. Anderson et al. [51] demonstrated that the body mass index (BMI) does not alter the diagnostic accuracy of a CT scan. CT appears therefore more reliable than ultrasonography in obese patients with the exception of children and pregnancy.

Patients with appendicitis are classified as uncomplicated or complicated appendicitis based upon pre-operative, intra-operative and/or histopathological findings. In this report, uncomplicated appendicitis has been defined as an inflamed appendix without signs of gangrene, perforation, intra-peritoneal purulent fluid, contained phlegmon or intra-abdominal abscess (IAA). Complicated appendicitis applies to all patients with either a gangrenous inflamed appendix with or without perforation, intra-abdominal abscess, peri-appendicular contained phlegmon or purulent free fluid. Classification is necessary as treatment strategies may differ.

### Uncomplicated appendicitis

Appendectomy is still considered to be the gold standard for uncomplicated appendicitis. Two main approaches to remove an inflamed appendix are available; the open approach (OA) or the laparoscopic approach (LA). In 2010, a large Cochrane review on 67 studies showed that LA significantly reduced the rate of surgical site infection (SSI) (OR 0.43; 95 % CI 0.34–0.54) but significantly increased the risk of an intra-abdominal abscess (IAA) (OR 1.77; 95 % CI 1.14–2.76) compared to the open approach [52]. It was stated that LA was associated with fewer superficial wound infections, less post-operative pain, shorter hospital stay and earlier return to work, but the higher rate of IAA raised concerns [52]. Ever since, inconsistent results have been reported regarding the potential higher incidence of IAA after LA [53–61]. Benefits of LA over OA reported in meta-analyses are: reduced incidence of SSI, post-operative and long-term bowel obstruction with better outcome in terms of shorter hospital stay, its diagnostic value, less pain, earlier return to work, earlier start of oral intake, improved scar and body satisfaction and fewer incisional hernias [54, 55, 58, 61–66]. Disadvantages besides the possible higher incidence of IAA are longer operating time and possibly increased costs [58, 63].

To reduce the surgical trauma even more, new treatment strategies have been introduced such as single-incision laparoscopic surgery (SILS) first reported by Pelosi et al. [67]. Since then, numerous studies (RCTs and SR) have been published on the potential advantages and disadvantages of the SILS technique. It can be concluded that SILS is associated with comparable post-operative morbidity rates compared to conventional LA [68–70]. The disadvantage is the fact that SILS is a more difficult technique as is reflected by the higher technical failure rate, longer operating time and conversion rate [71–78]. Main advantages of SILS would be less post-operative pain and better cosmetic outcomes, although inconsistent results have been reported [71, 75, 76, 79–81]. At the present time, evidence is lacking that SILS is superior to conventional LA [79, 82, 83]. SILS is, however, a safe and feasible alternative.

Recently, initial non-operative management of appendicitis has been investigated in the adult population. Five RCTs reported an effectiveness of 41–85 % at 1-year follow-up [84–88]. Meta-analyses of these studies revealed that non-operative treatment of acute appendicitis is less effective but could avoid surgery in 60–85 % of patients [89–94]. Opponents of this strategy raise concerns such as recurrent appendicitis, missing an underlying malignancy and progression of uncomplicated into complicated appendicitis. Due to the possible avoidance of surgery with an initial non-operative treatment strategy, morbidity was diminished [91, 93, 95]. However, both RCTs and meta-analyses showed significant heterogeneity of methodological quality, studies included and definitions of outcome parameters. Until higher qualitative evidence has been obtained regarding the potential benefits of initial non-operative management of acute appendicitis and the potential long-term effects have been investigated appropriately, appendectomy remains the gold standard in acute uncomplicated appendicitis.

### Complicated appendicitis

Due to the heterogeneity of the definitions used in the literature, it is difficult to draw firm conclusions regarding the treatment of complicated appendicitis. In 2013, Dimitriou published a retrospective cohort study on 150 patients with complicated appendicitis (defined as perforated with an abscess or peritonitis). They showed that LA reduced the incidence of SSI, number of reoperations and length of hospital stay as compared to OA with no difference in IAA rate [96]. A RCT encompassing 81 patients with clinically and histopathologically confirmed complicated appendicitis showed similar outcomes after OA and LA [97]. It should be noted, however, that the incidence of IAA after LA for patients with complicated appendicitis was reported to be higher in some studies. Tuggle and colleagues reported that LA in patients with complicated appendicitis was associated with an incidence of IAA of 6.7 versus 3.7 % in patients who underwent an open appendectomy [98]. The incidence of small bowel obstructions after LA is lower compared to OA (pooled odds ratio 0.44 [95 % CI 0.26–0.74] with large heterogeneity regarding follow-up period) [65].

In case of a contained phlegmon or abscess (peri-appendicular mass), some authors opt for non-operative treatment while others advocate aggressive operative treatment. In 2007, Andersson et al. [99] demonstrated that immediate surgical treatment of patient with an abscess or phlegmon was associated with higher morbidity compared to initial non-operative treatment (OR 3.3 95 % CI 1.9–5.6). Similis et al. showed in their meta-analysis of 17 studies regarding this specific patient group that non-operative treatment was associated with fewer complications (SSI, IAA and bowel obstructions). It must be mentioned that this meta-analysis was subject to large heterogeneity [100]. Recent cohort studies draw opposite conclusions [101, 102]. They opt for a more aggressive surgical approach at time of presentation in case of an appendicular mass or appendicular abscess, based upon the idea that there is a relative high failure rate for non-surgical treatment [101, 102]. In our opinion, with this new evidence, a new systematic review should be performed. Until then, initial non-operative treatment of an appendicular mass of appendicular abscess is the preferred treatment of choice. Although not covered in this consensus guideline, the value of interval appendectomy after initial non-operative treatment of an appendicular mass is still subject of debate. Some opt for an interval appendectomy based upon the chance of missing an underlying and untreated malignancy (incidence 6 %) and the chance of developing recurrent appendicitis (incidence 5–44 %) [101–103]. Both can be avoided with an interval appendectomy, although data are lacking on its benefits.

### Specific patient groups

#### Obese patients

Abdominal surgery in obese patients is challenging for both the anaesthesiologist and surgeon due to higher incidence of respiratory dysfunction, difficult access to the abdominal cavity, blurred anatomical landmarks and reduced working space in the abdominal cavity. Clarke et al. [104] performed a subgroup analysis among 37 patients (14 LA and 23 OA) with a BMI higher than 30 kg/m^2^ and reported similar morbidity after LA and OA [104]. This was confirmed by a meta-analysis, although a reduced length of hospital stay was noted after LA [105]. More recently, two recent meta-analyses showed a reduction of mortality and morbidity rates after LA [106, 107].

#### Pregnancy

Pregnancy induces anatomical and physiological changes that challenge the surgeon. The potential effects of carbon dioxide and increased abdominal pressure during LA on the foetus remain unclear. Loss of the foetus is most feared. In 2008, Walsh et al. [108] published a systematic review of 637 laparoscopic appendectomies in pregnant patients and noted foetal loss in approximately 6 % of the patients, with the highest incidence in patients with complicated appendicitis. Another review confirmed these findings and reported a nearly twofold increase of foetal loss in the LA group [109]. Both reviews, however, are mainly dominated by one study and based on low-grade evidence (retrospective studies with small numbers of patients) [108–110]. Recently, a review suggested that based upon the little available evidence no recommendation can be made regarding the preferred approach in pregnant patients [111]. More studies are necessary to ascertain the role of laparoscopic surgery during pregnancy. Until more evidence comes available, the surgical approach should be at the surgeon’s discretion. Based upon expert opinion, we recommend laparoscopy in case of sufficient experience. Although not supported by the literature, we strongly advise a multi-disciplinary approach to the pregnant patient with appendicitis [13, 54, 82, 111, 112].

#### Children

One meta-analysis included 107,624 children with both uncomplicated and complicated appendicitis [113]. Laparoscopic appendectomy in children with uncomplicated appendicitis LA was associated with a significant reduction of hospital stay with similar morbidity compared to open surgery. In children with complicated appendicitis, LA was associated with lower rates of morbidity, SSI, length of hospital admission and bowel obstruction. However, laparoscopic surgeries lasted longer and were followed by more intra-abdominal abscesses [113]. In more recent prospective cohort studies in children below 5 years of age, LA was associated with fewer complications [114]. Non-operative treatment of acute non-complicated appendicitis appears more promising in children than in adults [115, 116]

#### Elderly

Elderly patients have higher morbidity, reduced physiological reserves and impaired inflammatory responses, which increases their peri-operative risks. All studies of laparoscopic appendectomy in elderly support the use of laparoscopic surgery [117–121]. One meta-analysis, comprising more than 15,000 patients reported that LA reduced post-operative mortality (0.24; 95 % CI 0.15–0.37), post-operative complications (0.61; 95 % CI 0.50–0.73) and length of hospital stay (−0.51; 95 % CI −0.64 to −0.37) compared to OA (Tables 1, 2, 3, 4) [119].

**Table 1:**
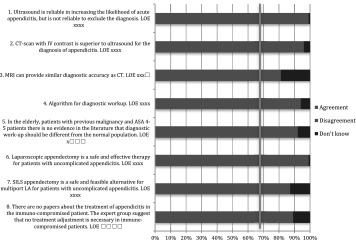
Pre-operative care: statements EAES meeting 2015

*LOE* level of evidence

X means present, Box means not present

**Table 2:**
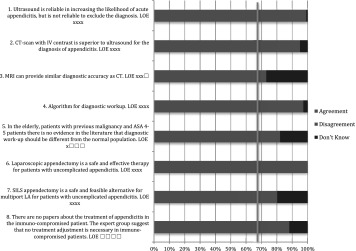
Pre-operative care: statements web survey

*LOE* level of evidence

X means present, Box means not present

**Table 3:**
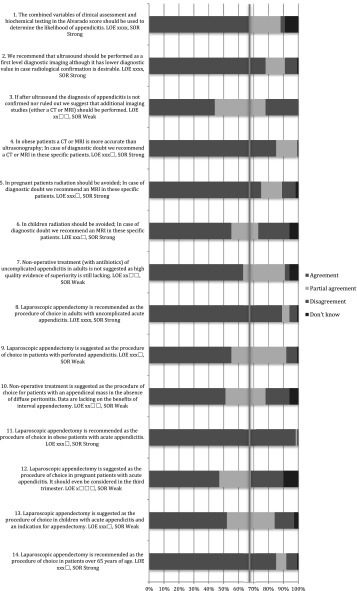
Pre-operative care: recommendations EAES meeting 2015

*LOE* level of evidence, *SOR* strength of recommendation

X means present, Box means not present

**Table 4:**
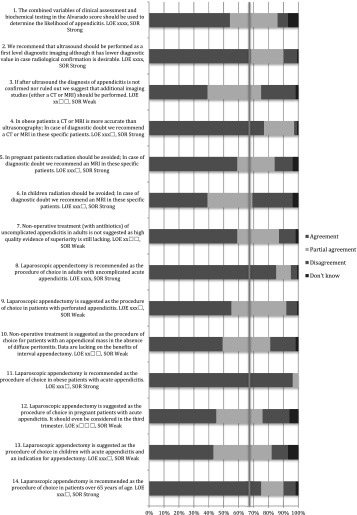
Pre-operative care: recommendations web survey

*LOE* level of evidence, *SOR* strength of recommendation

X means present, Box means not present

## Timing

Determining the best moment to perform surgery in case of acute appendicitis is of crucial importance [122, 123]. Acute appendicitis has been considered to be an irreversible progressive disease although recent studies have questioned this dogma [84, 89, 124]. Nowadays, the idea is endorsed that two types of appendicitis exist: uncomplicated (non-perforating) and complicated (perforating) appendicitis. The aetiology and pathogenesis of acute appendicitis remain largely unknown. Predicting a mild or fulminant course of appendicitis is not possible. Delaying an appendectomy increases the risk of perforated appendicitis, which is associated with higher incidence of short and long-term morbidity [125–127]. Hence, it is recommended to perform appendectomy as soon as possible. Although it should be noted that some studies have revealed that the clinical outcome was not affected by time to surgery (when surgery was performed within 12 h after presentation at the emergency department) [128, 129].

## Antibiotic prophylaxis

Antibiotic prophylaxis has been proven effective in prevention of superficial surgical site infections and intra-abdominal abscesses in patients with appendicitis [130–132]. Prophylaxis should be commenced at the time of establishing the diagnosis of acute appendicitis. The choice of antibiotics is dependent on the local microbiome and drug resistance pattern and is not influenced by age.

## Technique

Open access to the abdominal cavity as well as closed access using the Veress needle are accepted techniques to perform laparoscopy [133–135]. The debate on the preferred technique continues. However, in children, the majority of surgeons employs open establishment of a pneumoperitoneum.

The placement of the camera port and the work ports depend on the anatomy of the patient and preference of the operating surgeon. Primary principle of trocar placement in laparoscopy is that a triangular working space should be pursued.

## Intra-operative procedure

Increased employment of pre-operative radiologic testing (e.g. ultrasound, CT or MRI) in cases of suspected appendicitis has significantly reduced the incidence of a normal appearing appendix encountered during surgery [136]. Macroscopic distinction between a normal appendix and appendicitis during surgery can be difficult [137, 138]. The “gold standard” for defining appendicitis is histopathology. In some studies, histopathological assessment revealed abnormal findings in up to 26 % of macroscopically normal appearing appendices [139, 140]. Therefore, it is recommended to perform an appendectomy in case of a normal appearing appendix during surgery for suspected appendicitis.

Several studies have investigated the safety of different methods of securing the appendicular stump [82, 141–143]. None of the different closure methods has a clear advantage in case of a healthy appendix base. Stapler devices provide the most standardized and patent closure of the appendix base. Suturing of the appendix base provides sufficient closure as well, but is technically more demanding than other techniques [142]. In case of perforation of the appendicular base, clips or endoloops do not provide secure closure and staple devices or laparoscopic suturing is required [82].

Reduction of bacterial load by meticulous suction of intra-peritoneal fluids is advised [144–146]. The right paracolic and pelvic area should be inspected to leave no fluid collections behind. Irrigation of the intra-peritoneal space in case of perforated appendicitis seems to be contra-productive leading to a higher number of abscesses [144, 145]. It is believed that irrigation of the intra-peritoneal space leads to spreading of bacteria. Routine use of drains does not reduce the incidence of abscesses [145, 147]. Necessity of a drain for special indications is left to the discretion of the surgeon.

## Intra-operative unexpected findings

When an appendicular mass is encountered during surgery, one should restrain from continuing the operation. Continuation of the operation can necessitate bowel resection. Antibiotic treatment of phlegmon and drainage of any abscess should be performed [99, 148, 149].

The extent of surgical resection in case of suspected malignancy depends on the location and size of the appendicular mass [150–154]. Routine inclusion of the meso-appendix with the appendectomy is advised. Definitive histological findings determine whether an additional resection after total appendectomy is indicated. In cases of small neuroendocrine tumours (NET) or low-grade appendicular mucinous neoplasms (LAMN), a total meso-appendicular resection can be sufficient. In cases of a NET > 1 cm, LAMN grade 3–4 or an adenocarcinoma of the appendix, a formal right hemicolectomy is indicated to provide an oncologically sufficient resection. It is advised to perform a total meso-appendicular resection at the primary operation and an additional hemicolectomy at a later stage when indicated (Tables 5, 6, 7, 8) [150–154].

**Table 5:**
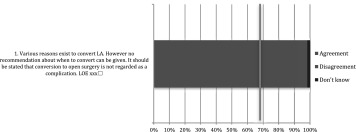
Operative care: statements EAES meeting 2015

*LOE* level of evidence

X means present, Box means not present

**Table 6:**
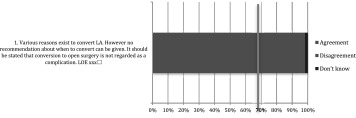
Operative care: statements web survey

*LOE* level of evidence

X means present, Box means not present

**Table 7:**
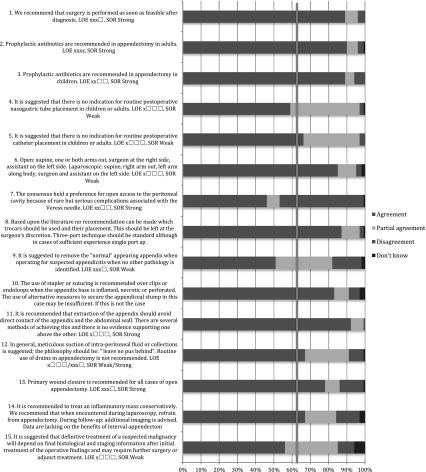
Operative care: recommendations EAES meeting 2015

*LOE* level of evidence, *SOR* strength of recommendation

X means present, Box means not present

**Table 8:**
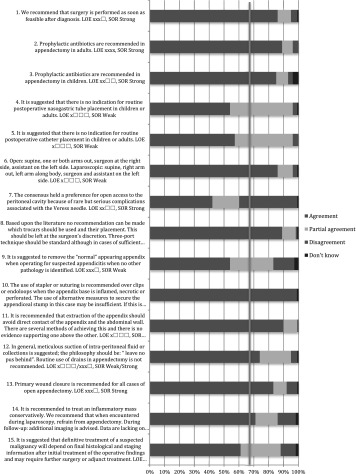
Operative care: recommendations web survey

*LOE* level of evidence, *SOR* strength of recommendation

X means present, Box means not present

## Post-operative antibiotics

The incidence of SSI after appendectomy has been reported to range from 0 to 11 % [155–164]. The severity of appendicitis strongly influences the risk of developing post-operative complications resulting in a substantially higher complication rate (up to 2–4 times) in patients with complicated appendicitis. In this specific group, post-operative administration of antibiotics significantly reduces the rate of SSI. In addition, to reduce bacteraemia and sepsis, these patients are uniformly treated with a course of post-operative antibiotics [155–158, 163]. In uncomplicated appendicitis, there is no evidence supporting routine administration of post-operative antibiotics. Therefore, only one pre-operative dose is advised [155–158].

Advice on type of antibiotics depends on local microbiome and resistance patterns and therefore should be left up to the discretion of the surgeon [159, 160]. Available evidence on duration of treatment is limited and mainly focused on children. However, there is no firm evidence on the duration (3, 5, 7, 10 days) and route of administration (usually intravenous administration for 48 h, then oral administration) [156, 157, 159, 161, 162].

## Post-operative complications

The incidence of post-operative complications ranges from 3.0 to 28.7 % [164–174]. Complications include small bowel obstruction (0–1.9 %.), SSI (1.2–12.0 %), IAA (1.6–8 %), stump leakage and stump appendicitis [164–174]. Literature suggests a higher rate of complications in complicated appendicitis [166, 167, 171, 175].

Literature on stump leakage and stump appendicitis is limited, and no exact incidences have been reported in the literature, although it is assumed that it is more common in patients with complicated appendicitis and after OA [176]. A recommendation to avoid stump leakage or stump appendicitis is to resect the appendix as a whole [176]. Therefore, the stump should be no longer than 0.5 cm and caecal taenia should be followed onto the appendix at removal to ensure complete resection. Stump appendicitis is significantly more associated with perforation, as diagnosis is delayed by misled attention. This is caused by the assumption that the appendix as a whole is resected. Prevention is crucial. In case of timely diagnosis, stump resection with laparoscopic or open approach is feasible. In case of perforation, extended bowel resection is usually required [176].

In the initial management of IAA after appendectomy conservative measures (i.e. non-operative with antibiotics) are effective in most patients. However, in case of lack of improvement or deterioration, a more invasive strategy should be applied (percutaneous drainage or surgical (laparoscopic) drainage) [177–179].

## Post-operative care

The use of prophylactic anti-emetics diminishes the incidence of post-operative nausea and vomiting. Increasing the diet is best determined by the patient’s ability to tolerate oral intake. There is no evidence that a liberal diet causes complications in the post-operative period [164, 180].

Post-operative pain management should follow local protocol for pain management after abdominal surgery. Post-operative analgesia with PCA provides effective and safe pain relief in children and adults and is less time costly [181]. Recently positive results have been published regarding the pre-emptive incision site infiltration with a local anaesthetic. Studies demonstrated that this decreases the total opioid consumption and lowers pain score experienced by patients in the first 24 h after surgery [182–184].

## Pathology

Carcinoid is the most commonly found neoplasm in appendectomy specimens at an incidence between 0.13 and 2.4 % [185–190]. Other unexpected findings can be encountered in 1.4–2.4 % of patients, including: diverticulitis (1.2 %), tuberculosis appendix 0.08 %, endometriosis (3.6 %), adenocarcinoma (<1 %) and mucinous cystadenoma (0.2–0.6 %) [191–194].

Treatment of unexpected findings ranges from no further surgical treatment, to right hemi colectomy and even hyperthermic intra-peritoneal chemotherapy (HIPEC) in some cases [195–197]. Even though the incidence of unexpected findings seems low, the actual number of patients is significant and correct diagnosis is crucial for adequate treatment (Tables 9, 10, 11, 12) [198–202].

**Table 9:**
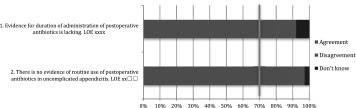
After care: statements EAES meeting 2015

*LOE* level of evidence

X means present, Box means not present

**Table 10:**
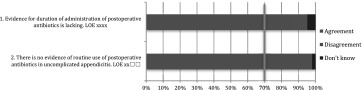
After care: statements web survey

*LOE* level of evidence

X means present, Box means not present

**Table 11:**
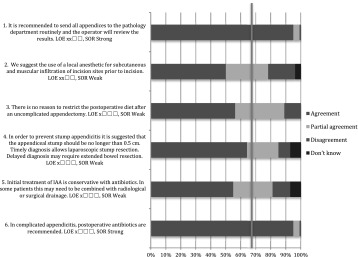
After care: recommendations EAES meeting

*LOE* level of evidence, *SOR* strength of recommendation

X means present, Box means not present

**Table 12:**
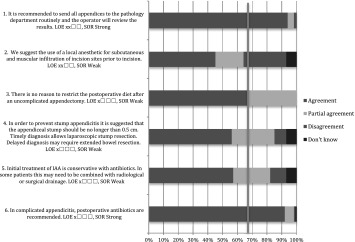
After care: recommendations web survey

*LOE* level of evidence, *SOR* strength of recommendation

X means present, Box means not present

## Discussion

This EAES consensus development conference regarding the diagnosis and management of acute appendicitis resulted in 46 statements and recommendations based upon the available evidence. Results from this meeting led to this paper, which can be used as a guideline for surgeons treating patients with appendicitis. Local guidelines, national guidelines and guidelines from scientific communities regarding appendicitis were available but showed great heterogeneity [13, 14, 203]. With this consensus meeting, we managed to gather experts from different European nations to compare and debate management of patients with acute appendicitis. This led to a consensus meeting in which 41 of the 46 statements and the majority of the members of the EAES supported recommendations. The transfer of knowledge between the member countries, the opportunity to discuss views and above all, the creation of a widely supported paper appears valuable.

Our list of topics was created by the coordinating team and expert panel and was thought to cover the most important topics in the field of acute appendicitis. Despite local differences, the general idea within the consensus group on the management of patients with acute appendicitis was comparable. In some cases, differences of treatment strategies between members of the expert panel were due to available surgical supplies and finances. This is reflected for instance on the statements and recommendations regarding SILS and MRI. However, we want to emphasize that in defining statements we refrained from stating specific procedures. We rather stated the general principles to follow. In this way, the results from this consensus guideline can also be applied in areas with limited resources.

The methodology of a consensus guideline is always subject to discussion. In the literature, there are several ways to conduct consensus conferences [20, 204–206]. However, not one was suited for our situation. It was therefore decided to modify the Delphi method, as described in the method section, in order to systematically evaluate each statement and recommendation [20–22]. We decided to finalize only those statements and recommendation with 70 % or more consensus, which is the arbitrary cut-off value we selected. The results of both the web-based survey and the live voting at the EAES conference in Bucharest are presented independently rather than combined to rule out any bias. As expected, small differences were noted between the several voting rounds. Although supported by the experts, some statements and recommendations were not supported by the scientific community in both the web survey as in the Bucharest meeting. The topics that were not supported were on accuracy of MRI compared to CT, the application of SILS, extensive work-up in the elderly and treatment strategy for immune compromised patients and the open access to the peritoneal cavity. Explanations for these discrepancies might be related to local habits, experience and financial situation. Of more interest are the discrepancies noted between the outcome in the web survey and during the Bucharest meeting. Discrepancies were noted on the topic of MRI application in children, the preferred approach in pregnant patients and the use of local anaesthetics prior to incision. This can again be explained by the fact that local habits, experience, composition of the voting public and financial situation might influence the outcome. The question was raised if the web survey alone would be sufficient to reach a consensus for future meetings. Limiting a consensus meeting to only the web survey would limit the time as well as the costs involved. Moreover, a higher percentage of surgeons participated in the web survey. In our opinion, however, the integration of an actual face-to-face meeting in the consensus methodology raises more awareness, provides an opportunity to discuss views and encourages the transfer of knowledge eventually leading to the creation of a widely supported paper.

The literature review was ended in December 2014. No studies after that were integrated for the consensus meeting as this was decided in our methodology. Therefore, new studies might have been conducted on some topics. Future research should be focused on the laparoscopic appendectomy in pregnant patients, elucidating the value of MRI in specific patient groups, evaluating the outcomes of initial non-operative treatment for both uncomplicated and complicated appendicitis, specific patient groups and the need for interval appendectomy. We therefore propose that these statements are updated on a regular basis.

Although some limitations can be identified in our methodology, we have integrated a new systematic method for a consensus meeting. In our opinion, this is the way forward and we need to efflorescence this method. Reproducibility, involving members of the scientific community and applicability are key components of a consensus meeting. We believe that only after evaluation of the general opinion within the EAES such guidelines should be put into order.

In conclusion, the consensus meeting of the EAES resulted in several statements and recommendations regarding the diagnosis and management of appendicitis based upon available evidence and expert opinion and was supported by the European surgical community. It provides guidance to surgeons and surgical residents facing patients with acute appendicitis.

## Electronic supplementary material

Below is the link to the electronic supplementary material.

Supplementary material 1 (DOCX 80 kb)

Supplementary material 2 (DOCX 32 kb)

Supplementary material 3 (DOCX 87 kb)
