# Supplementary material for: Diagnosis and management of acute appendicitis. EAES consensus development conference 2015
Source: Surg Endosc. 2016 Sep 22;30(11):4668–90. doi: 10.1007/s00464-016-5245-7 (PMC5082605; doi:10.1007/s00464-016-5245-7)
Supplement: Supplementary file 1 — Supplementary material 1 (DOCX 80 kb) [file 464_2016_5245_MOESM1_ESM.docx]

Appendix #1

Topics discussed during the Amsterdam Meeting including # of statements and recommendations per topic

| Main topic |  | Number of statements/recommendations |
| --- | --- | --- |
| Pre-operative care |  |  |
|  | Incidence & prevalence | 4 |
|  | Diagnostic work-up | 12 |
|  | Treatment indications | 12 |
| Operative care |  |  |
|  | General aspects | 2 |
|  | Before surgery | 7 |
|  | Intra-operative | 12 |
| After care |  |  |
|  | Postoperative antibiotics | 5 |
|  | Postoperative complications & care | 13 |
|  | Pathology | 8 |
|  |  |  |
| Total |  | **75** |
