# Supplementary material for: Diagnosis and management of acute appendicitis. EAES consensus development conference 2015
Source: Surg Endosc. 2016 Sep 22;30(11):4668–90. doi: 10.1007/s00464-016-5245-7 (PMC5082605; doi:10.1007/s00464-016-5245-7)
Supplement: Supplementary file 2 — Supplementary material 2 (DOCX 32 kb) [file 464_2016_5245_MOESM2_ESM.docx]

# Appendix #2

# Literature search

# Primary search # 1: yielded 8147 articles

# Additional search #31: yielded additional 4985 articles

# Total hits: 13,132 articles

| **Search** | **Query** | **Items found** |
| --- | --- | --- |
| [#31](http://www.ncbi.nlm.nih.gov/pubmed/advanced) | Search **(#11 OR #18 OR #21 OR #24 OR #27)** | [4985](http://www.ncbi.nlm.nih.gov/pubmed/?cmd=HistorySearch&querykey=31) |
| [#30](http://www.ncbi.nlm.nih.gov/pubmed/advanced) | Search **((#2 AND #28) NOT #1)** | [13470](http://www.ncbi.nlm.nih.gov/pubmed/?cmd=HistorySearch&querykey=30) |
| [#29](http://www.ncbi.nlm.nih.gov/pubmed/advanced) | Search **(#2 AND #28)** | [17475](http://www.ncbi.nlm.nih.gov/pubmed/?cmd=HistorySearch&querykey=29) |
| [#28](http://www.ncbi.nlm.nih.gov/pubmed/advanced) | Search **(diagnosis[sh] OR ultrasonography[sh] OR diagnos*[tiab] OR ultrasound[tiab] OR computed tomograph*[tiab] OR diagnos*[ot] OR ultrasound[ot] OR computed tomograph*[ot])** | [5293263](http://www.ncbi.nlm.nih.gov/pubmed/?cmd=HistorySearch&querykey=28) |
| [#27](http://www.ncbi.nlm.nih.gov/pubmed/advanced) | Search **((#2 AND #25) NOT #1)** | [633](http://www.ncbi.nlm.nih.gov/pubmed/?cmd=HistorySearch&querykey=27) |
| [#26](http://www.ncbi.nlm.nih.gov/pubmed/advanced) | Search **(#2 AND #25)** | [1075](http://www.ncbi.nlm.nih.gov/pubmed/?cmd=HistorySearch&querykey=26) |
| [#25](http://www.ncbi.nlm.nih.gov/pubmed/advanced) | Search **(Cost-Benefit Analysis[mh] OR Hospital Costs[mh] OR economics[sh] OR cost[tiab] OR costs[tiab] OR financial*[tiab] OR cost[ot] OR costs[ot] OR financial*[ot])** | [614869](http://www.ncbi.nlm.nih.gov/pubmed/?cmd=HistorySearch&querykey=25) |
| [#24](http://www.ncbi.nlm.nih.gov/pubmed/advanced) | Search **((#2 AND #22) NOT #1)** | [324](http://www.ncbi.nlm.nih.gov/pubmed/?cmd=HistorySearch&querykey=24) |
| [#23](http://www.ncbi.nlm.nih.gov/pubmed/advanced) | Search **(#2 AND #22)** | [631](http://www.ncbi.nlm.nih.gov/pubmed/?cmd=HistorySearch&querykey=23) |
| [#22](http://www.ncbi.nlm.nih.gov/pubmed/advanced) | Search **(Leukocyte Count[mh] OR white blood cell[tiab] OR wbc[tiab] OR white blood cell[ot] OR wbc[ot])** | [98248](http://www.ncbi.nlm.nih.gov/pubmed/?cmd=HistorySearch&querykey=22) |
| [#21](http://www.ncbi.nlm.nih.gov/pubmed/advanced) | Search **((#2 AND #19) NOT #1)** | [1089](http://www.ncbi.nlm.nih.gov/pubmed/?cmd=HistorySearch&querykey=21) |
| [#20](http://www.ncbi.nlm.nih.gov/pubmed/advanced) | Search **(#2 AND #19)** | [2284](http://www.ncbi.nlm.nih.gov/pubmed/?cmd=HistorySearch&querykey=20) |
| [#19](http://www.ncbi.nlm.nih.gov/pubmed/advanced) | Search **(“Anti-Bacterial Agents”[mh] OR “Anti-Bacterial Agents”[pa] OR antibiotic*[tiab] OR cephalosporin*[tiab] OR metronidazol*[tiab] OR mesalazin*[tiab] OR antibiotic*[ot] OR cephalosporin*[ot] OR metronidazol*[ot] OR mesalazin*[ot])** | [678761](http://www.ncbi.nlm.nih.gov/pubmed/?cmd=HistorySearch&querykey=19) |
| [#18](http://www.ncbi.nlm.nih.gov/pubmed/advanced) | Search **((#2 AND #16) NOT #1)** | [1118](http://www.ncbi.nlm.nih.gov/pubmed/?cmd=HistorySearch&querykey=18) |
| [#17](http://www.ncbi.nlm.nih.gov/pubmed/advanced) | Search **(#2 AND #16)** | [1773](http://www.ncbi.nlm.nih.gov/pubmed/?cmd=HistorySearch&querykey=17) |
| [#16](http://www.ncbi.nlm.nih.gov/pubmed/advanced) | Search **(“Aged, 80 and over”[mh] OR elderly[tiab] OR elderly[ot])** | [754294](http://www.ncbi.nlm.nih.gov/pubmed/?cmd=HistorySearch&querykey=16) |
| [#15](http://www.ncbi.nlm.nih.gov/pubmed/advanced) | Search **((#2 AND #12) NOT #1)** | [8039](http://www.ncbi.nlm.nih.gov/pubmed/?cmd=HistorySearch&querykey=15) |
| [#13](http://www.ncbi.nlm.nih.gov/pubmed/advanced) | Search **(#2 AND #12)** | [11528](http://www.ncbi.nlm.nih.gov/pubmed/?cmd=HistorySearch&querykey=13) |
| [#12](http://www.ncbi.nlm.nih.gov/pubmed/advanced) | Search **(child*[tw] OR schoolchild*[tw] OR infan*[tw] OR adolescen*[tw] OR pediatri*[tw] OR paediatr*[tw] OR neonat*[tw] OR boy[tw] OR boys[tw] OR boyhood[tw] OR girl[tw] OR girls[tw] OR girlhood[tw] OR youth[tw] OR youths[tw] OR baby[tw] OR babies[tw] OR toddler*[tw] OR "Mental Disorders Diagnosed in Childhood"[MeSH] OR teen[tw] OR teens[tw] OR teenager*[tw] OR newborn*[tw] OR postneonat*[tw] OR postnat*[tw] OR perinat*[tw] OR puberty[tw] OR preschool*[tw] OR suckling*[tw] OR picu[tw] OR nicu[tw] OR "Arthritis, Juvenile"[Mesh] OR "Myoclonic Epilepsy, Juvenile"[Mesh] OR "Leukemia, Myelomonocytic, Juvenile"[Mesh] OR "Xanthogranuloma, Juvenile"[Mesh] OR "Juvenile Delinquency"[Mesh] OR "Corneal Dystrophy, Juvenile Epithelial of Meesmann"[Mesh])** | [3557608](http://www.ncbi.nlm.nih.gov/pubmed/?cmd=HistorySearch&querykey=12) |
| [#11](http://www.ncbi.nlm.nih.gov/pubmed/advanced) | Search **((#2 AND #9) NOT #1)** | [2571](http://www.ncbi.nlm.nih.gov/pubmed/?cmd=HistorySearch&querykey=11) |
| [#10](http://www.ncbi.nlm.nih.gov/pubmed/advanced) | Search **(#2 AND #9)** | [3860](http://www.ncbi.nlm.nih.gov/pubmed/?cmd=HistorySearch&querykey=10) |
| [#9](http://www.ncbi.nlm.nih.gov/pubmed/advanced) | Search **(Laparoscopy[mh] OR laparoscop*[tiab] OR laparoscop*[ot])** | [99628](http://www.ncbi.nlm.nih.gov/pubmed/?cmd=HistorySearch&querykey=9) |
| [#8](http://www.ncbi.nlm.nih.gov/pubmed/advanced) | Search **((#2 AND #6) NOT #1)** | [6022](http://www.ncbi.nlm.nih.gov/pubmed/?cmd=HistorySearch&querykey=8) |
| [#7](http://www.ncbi.nlm.nih.gov/pubmed/advanced) | Search **(#2 AND #6)** | [9793](http://www.ncbi.nlm.nih.gov/pubmed/?cmd=HistorySearch&querykey=7) |
| [#6](http://www.ncbi.nlm.nih.gov/pubmed/advanced) | Search **("Epidemiologic Studies"[Mesh] OR Observational Study[pt] OR cohort[tiab] OR (case[tiab] AND (control[tiab] OR controll*[tiab] OR comparison[tiab] OR referent[tiab])) OR risk[tiab] OR causation[tiab] OR causal[tiab] OR "odds ratio"[tiab] OR etiol*[tiab] OR aetiol*[tiab] OR "natural history"[tiab] OR predict*[tiab] OR prognos*[tiab] OR outcome[tiab] OR course[tiab] OR retrospecti*[tiab] OR multivariate analys*[tiab] OR prospecti*[tiab] OR cohort[ot] OR (case[ot] AND (control[ot] OR controll*[ot] OR comparison[ot] OR referent[ot])) OR risk[ot] OR causation[ot] OR causal[ot] OR "odds ratio"[ot] OR etiol*[ot] OR aetiol*[ot] OR "natural history"[ot] OR predict*[ot] OR prognos*[ot] OR outcome[ot] OR course[ot] OR retrospecti*[ot] OR multivariate analys*[ot] OR prospecti*[ot])** | [4629507](http://www.ncbi.nlm.nih.gov/pubmed/?cmd=HistorySearch&querykey=6) |
| [#5](http://www.ncbi.nlm.nih.gov/pubmed/advanced) | Search **(#2 AND #3 NOT #1)** | [16857](http://www.ncbi.nlm.nih.gov/pubmed/?cmd=HistorySearch&querykey=5) |
| [#4](http://www.ncbi.nlm.nih.gov/pubmed/advanced) | Search **(#2 AND #3)** | [23253](http://www.ncbi.nlm.nih.gov/pubmed/?cmd=HistorySearch&querykey=4) |
| [#3](http://www.ncbi.nlm.nih.gov/pubmed/advanced) | Search **(Intraabdominal Infections[mh] OR Patient Readmission[mh] OR Intestinal Perforation[mh] OR Abdominal Abscess[mh] OR Recurrence[mh] OR Treatment Failure[mh] OR Treatment Outcome[mh] OR Length of Stay[mh] OR complications[sh] OR Operative Time[mh] OR uncomplicat*[tiab] OR complicat*[tiab] OR postoperative*[tiab] OR mortality[tiab] OR readmission*[tiab] OR reoperation*[tiab] OR operative time[tiab] OR length of stay*[tiab] OR abscess*[tiab] OR wound infection*[tiab] OR incisional hernia*[tiab] OR comorbidit*[tiab] OR recurren*[tiab] OR perforat*[tiab] OR bleeding*[tiab] OR hospital stay*[tiab] OR uncomplicat*[ot] OR complicat*[ot] OR postoperative*[ot] OR mortality[ot] OR readmission*[ot] OR reoperation*[ot] OR operative time[ot] OR length of stay*[ot] OR abscess*[ot] OR wound infection*[ot] OR incisional hernia*[ot] OR comorbidit*[ot] OR recurren*[ot] OR perforat*[ot] OR bleeding*[ot] OR hospital stay*[ot])** | [3792009](http://www.ncbi.nlm.nih.gov/pubmed/?cmd=HistorySearch&querykey=3) |
| [#2](http://www.ncbi.nlm.nih.gov/pubmed/advanced) | Search **("Appendix"[Mesh] OR appendix[tiab] OR appendix[ot] OR "Appendicitis"[Mesh] OR "Appendectomy"[Mesh] OR appendicit*[tiab] OR appendicit*[ot] OR appendectom*[tiab] OR appendectom*[ot] OR appendicectom*[tiab] OR appendicectom*[ot])** | [34467](http://www.ncbi.nlm.nih.gov/pubmed/?cmd=HistorySearch&querykey=2) |
| [#1](http://www.ncbi.nlm.nih.gov/pubmed/advanced) | Search **((((((((((((((((((((("Appendicitis"[Mesh] OR "Appendectomy"[Mesh] OR appendicit*[tiab] OR appendicit*[ot] OR appendectom*[tiab] OR appendectom*[ot] OR appendicectom*[tiab] OR appendicectom*[ot]) AND (therapy/narrow[filter] OR systematic[sb]))) OR ((Diagnosis/Narrow[filter] OR systematic[sb] OR therapy/narrow[filter]) AND ((("Appendix"[Mesh] OR appendix[tiab] OR appendix[ot] OR "Appendicitis"[Mesh] OR "Appendectomy"[Mesh] OR appendicit*[tiab] OR appendicit*[ot] OR appendectom*[tiab] OR appendectom*[ot] OR appendicectom*[tiab] OR appendicectom*[ot]) AND ("Diagnosis"[Mesh] OR "diagnosis"[Subheading] OR "Diagnostic Imaging"[Mesh] OR "Physical Examination"[Mesh] OR "Leukocytosis"[Mesh] OR "C-Reactive Protein"[Mesh] OR "Laparoscopy"[Mesh] OR "Magnetic Resonance Imaging"[Mesh] OR ("magnetic resonance"[tiab] AND (image[tiab] OR images[tiab] OR imaging[tiab])) OR mri[tiab] OR mris[tiab] OR nmr[tiab] OR mra[tiab] OR mras[tiab] OR zeugmatograph*[tiab] OR "mr tomography"[tiab] OR "mr tomographies"[tiab] OR "mr tomographic"[tiab] OR "proton spin"[tiab] OR ((magneti*[tiab] OR "chemical shift"[tiab]) AND imaging[tiab]) OR fmri[tiab] OR fmris[tiab] OR "Ultrasonography"[Mesh] OR "ultrasonography "[Subheading] OR ultraso*[tiab] OR sonograph*[tiab] OR echograph*[tiab] OR echocardiograph*[tiab] OR echotomograph*[tiab]))))) OR ((Diagnosis/Narrow[filter]) AND ((("Appendix"[Mesh] OR appendix[tiab] OR appendix[ot] OR "Appendicitis"[Mesh] OR "Appendectomy"[Mesh] OR appendicit*[tiab] OR appendicit*[ot] OR appendectom*[tiab] OR appendectom*[ot] OR appendicectom*[tiab] OR appendicectom*[ot]) AND ("Diagnosis"[Mesh] OR "diagnosis"[Subheading] OR "Diagnostic Imaging"[Mesh] OR "Physical Examination"[Mesh] OR "Leukocytosis"[Mesh] OR "C-Reactive Protein"[Mesh] OR "Laparoscopy"[Mesh] OR "Magnetic Resonance Imaging"[Mesh] OR ("magnetic resonance"[tiab] AND (image[tiab] OR images[tiab] OR imaging[tiab])) OR mri[tiab] OR mris[tiab] OR nmr[tiab] OR mra[tiab] OR mras[tiab] OR zeugmatograph*[tiab] OR "mr tomography"[tiab] OR "mr tomographies"[tiab] OR "mr tomographic"[tiab] OR "proton spin"[tiab] OR ((magneti*[tiab] OR "chemical shift"[tiab]) AND imaging[tiab]) OR fmri[tiab] OR fmris[tiab] OR "Ultrasonography"[Mesh] OR "ultrasonography "[Subheading] OR ultraso*[tiab] OR sonograph*[tiab] OR echograph*[tiab] OR echocardiograph*[tiab] OR echotomograph*[tiab]))))) OR (((systematic[sb] OR therapy/narrow[filter]) AND ((("Appendix"[Mesh] OR appendix[tiab] OR appendix[ot] OR "Appendicitis"[Mesh] OR "Appendectomy"[Mesh] OR appendicit*[tiab] OR appendicit*[ot] OR appendectom*[tiab] OR appendectom*[ot] OR appendicectom*[tiab] OR appendicectom*[ot]) AND ("Diagnosis"[Mesh] OR "diagnosis"[Subheading] OR "Diagnostic Imaging"[Mesh] OR "Physical Examination"[Mesh] OR "Leukocytosis"[Mesh] OR "C-Reactive Protein"[Mesh] OR "Laparoscopy"[Mesh] OR "Magnetic Resonance Imaging"[Mesh] OR ("magnetic resonance"[tiab] AND (image[tiab] OR images[tiab] OR imaging[tiab])) OR mri[tiab] OR mris[tiab] OR nmr[tiab] OR mra[tiab] OR mras[tiab] OR zeugmatograph*[tiab] OR "mr tomography"[tiab] OR "mr tomographies"[tiab] OR "mr tomographic"[tiab] OR "proton spin"[tiab] OR ((magneti*[tiab] OR "chemical shift"[tiab]) AND imaging[tiab]) OR fmri[tiab] OR fmris[tiab] OR "Ultrasonography"[Mesh] OR "ultrasonography "[Subheading] OR ultraso*[tiab] OR sonograph*[tiab] OR echograph*[tiab] OR echocardiograph*[tiab] OR echotomograph*[tiab])))) NOT ((Diagnosis/Narrow[filter]) AND ((("Appendix"[Mesh] OR appendix[tiab] OR appendix[ot] OR "Appendicitis"[Mesh] OR "Appendectomy"[Mesh] OR appendicit*[tiab] OR appendicit*[ot] OR appendectom*[tiab] OR appendectom*[ot] OR appendicectom*[tiab] OR appendicectom*[ot]) AND ("Diagnosis"[Mesh] OR "diagnosis"[Subheading] OR "Diagnostic Imaging"[Mesh] OR "Physical Examination"[Mesh] OR "Leukocytosis"[Mesh] OR "C-Reactive Protein"[Mesh] OR "Laparoscopy"[Mesh] OR "Magnetic Resonance Imaging"[Mesh] OR ("magnetic resonance"[tiab] AND (image[tiab] OR images[tiab] OR imaging[tiab])) OR mri[tiab] OR mris[tiab] OR nmr[tiab] OR mra[tiab] OR mras[tiab] OR zeugmatograph*[tiab] OR "mr tomography"[tiab] OR "mr tomographies"[tiab] OR "mr tomographic"[tiab] OR "proton spin"[tiab] OR ((magneti*[tiab] OR "chemical shift"[tiab]) AND imaging[tiab]) OR fmri[tiab] OR fmris[tiab] OR "Ultrasonography"[Mesh] OR "ultrasonography "[Subheading] OR ultraso*[tiab] OR sonograph*[tiab] OR echograph*[tiab] OR echocardiograph*[tiab] OR echotomograph*[tiab])))))) OR (((((review*[tiab] OR search*[tiab] OR survey*[tiab] OR handsearch*[tiab] OR hand-search*[tiab]) AND (databa*[tiab] OR data-ba*[tiab] OR bibliograph*[tiab] OR electronic*[tiab] OR medline*[tiab] OR pubmed*[tiab] OR embase*[tiab] OR Cochrane[tiab] OR cinahl[tiab] OR psycinfo[tiab] OR psychinfo[tiab] OR cinhal[tiab] OR "web of science"[tiab] OR "web of knowledge"[tiab] OR ebsco[tiab] OR ovid[tiab] OR mrct[tiab] OR metaregist*[tiab] OR meta-regist*[tiab] OR ((predetermined[tiab] OR pre-determined[tiab]) AND criteri*[tiab]) OR apprais*[tiab] OR inclusion criteri*[tiab] OR exclusion criteri*[tiab]) OR (review[pt] AND systemat*[tiab]) OR "systematic review"[tiab] OR "systematic literature"[tiab] OR "integrative review"[tiab] OR "integrative literature"[tiab] OR "evidence-based review"[tiab] OR "evidence-based overview"[tiab] OR "evidence-based literature"[tiab] OR "evidence-based survey"[tiab] OR "literature search"[tiab] OR ((systemat*[ti] OR evidence-based[ti]) AND (review*[ti] OR literature[ti] OR overview[ti] OR survey[ti])) OR "data synthesis"[tiab] OR "evidence synthesis"[tiab] OR "data extraction"[tiab] OR "data source"[tiab] OR "data sources"[tiab] OR "study selection"[tiab] OR "methodological quality"[tiab] OR "methodologic quality"[tiab] OR cochrane database syst rev[ta] OR meta-analy*[tiab] OR metaanaly*[tiab] OR metanaly*[tiab] OR meta-analysis[pt] OR meta-synthesis[tiab] OR metasynthesis[tiab] OR meta-study[tiab] OR metastudy[tiab] OR metaethnograph*[tiab] OR meta-ethnograph*[tiab] OR Technology Assessment, Biomedical[mh] OR hta[tiab] OR health technol assess[ta] OR evid rep technol assess summ[ta] OR health technology assessment[tiab]) OR ((review*[ot] OR search*[ot] OR survey*[ot] OR handsearch*[ot] OR hand-search*[ot]) AND (databa*[ot] OR data-ba*[ot] OR bibliograph*[ot] OR electronic*[ot] OR medline*[ot] OR pubmed*[ot] OR embase*[ot] OR cochrane[ot] OR cinahl[ot] OR psycinfo[ot] OR psychinfo[ot] OR cinhal[ot] OR "web of science"[ot] OR "web of knowledge"[ot] OR ebsco[ot] OR ovid[ot] OR mrct[ot] OR metaregist*[ot] OR meta-regist*[ot] OR ((predetermined[ot] OR pre-determined[ot]) AND criteri*[ot]) OR apprais*[ot] OR inclusion criteri*[ot] OR exclusion criteri*[ot]) OR (review[pt] AND systemat*[ot]) OR "systematic review"[ot] OR "systematic literature"[ot] OR "integrative review"[ot] OR "integrative literature"[ot] OR "evidence-based review"[ot] OR "evidence-based overview"[ot] OR "evidence-based literature"[ot] OR "evidence-based survey"[ot] OR "literature search"[ot] OR ((systemat*[ti] OR evidence-based[ti]) AND (review*[ti] OR literature[ti] OR overview[ti] OR survey[ti])) OR "data synthesis"[ot] OR "evidence synthesis"[ot] OR "data extraction"[ot] OR "data source"[ot] OR "data sources"[ot] OR "study selection"[ot] OR "methodological quality"[ot] OR "methodologic quality"[ot] OR meta-analy*[ot] OR metaanaly*[ot] OR metanaly*[ot] OR meta-analysis[pt] OR meta-synthesis[ot] OR metasynthesis[ot] OR meta-study[ot] OR metastudy[ot] OR metaethnograph*[ot] OR meta-ethnograph*[ot] OR hta[ot] OR health technology assessment[ot])) AND ("Appendix"[Mesh] OR appendix[tiab] OR appendix[ot] OR "Appendicitis"[Mesh] OR "Appendectomy"[Mesh] OR appendicit*[tiab] OR appendicit*[ot] OR appendectom*[tiab] OR appendectom*[ot] OR appendicectom*[tiab] OR appendicectom*[ot])) NOT (((Diagnosis/Narrow[filter]) AND ((("Appendix"[Mesh] OR appendix[tiab] OR appendix[ot] OR "Appendicitis"[Mesh] OR "Appendectomy"[Mesh] OR appendicit*[tiab] OR appendicit*[ot] OR appendectom*[tiab] OR appendectom*[ot] OR appendicectom*[tiab] OR appendicectom*[ot]) AND ("Diagnosis"[Mesh] OR "diagnosis"[Subheading] OR "Diagnostic Imaging"[Mesh] OR "Physical Examination"[Mesh] OR "Leukocytosis"[Mesh] OR "C-Reactive Protein"[Mesh] OR "Laparoscopy"[Mesh] OR "Magnetic Resonance Imaging"[Mesh] OR ("magnetic resonance"[tiab] AND (image[tiab] OR images[tiab] OR imaging[tiab])) OR mri[tiab] OR mris[tiab] OR nmr[tiab] OR mra[tiab] OR mras[tiab] OR zeugmatograph*[tiab] OR "mr tomography"[tiab] OR "mr tomographies"[tiab] OR "mr tomographic"[tiab] OR "proton spin"[tiab] OR ((magneti*[tiab] OR "chemical shift"[tiab]) AND imaging[tiab]) OR fmri[tiab] OR fmris[tiab] OR "Ultrasonography"[Mesh] OR "ultrasonography "[Subheading] OR ultraso*[tiab] OR sonograph*[tiab] OR echograph*[tiab] OR echocardiograph*[tiab] OR echotomograph*[tiab])))) OR (((systematic[sb] OR therapy/narrow[filter]) AND ((("Appendix"[Mesh] OR appendix[tiab] OR appendix[ot] OR "Appendicitis"[Mesh] OR "Appendectomy"[Mesh] OR appendicit*[tiab] OR appendicit*[ot] OR appendectom*[tiab] OR appendectom*[ot] OR appendicectom*[tiab] OR appendicectom*[ot]) AND ("Diagnosis"[Mesh] OR "diagnosis"[Subheading] OR "Diagnostic Imaging"[Mesh] OR "Physical Examination"[Mesh] OR "Leukocytosis"[Mesh] OR "C-Reactive Protein"[Mesh] OR "Laparoscopy"[Mesh] OR "Magnetic Resonance Imaging"[Mesh] OR ("magnetic resonance"[tiab] AND (image[tiab] OR images[tiab] OR imaging[tiab])) OR mri[tiab] OR mris[tiab] OR nmr[tiab] OR mra[tiab] OR mras[tiab] OR zeugmatograph*[tiab] OR "mr tomography"[tiab] OR "mr tomographies"[tiab] OR "mr tomographic"[tiab] OR "proton spin"[tiab] OR ((magneti*[tiab] OR "chemical shift"[tiab]) AND imaging[tiab]) OR fmri[tiab] OR fmris[tiab] OR "Ultrasonography"[Mesh] OR "ultrasonography "[Subheading] OR ultraso*[tiab] OR sonograph*[tiab] OR echograph*[tiab] OR echocardiograph*[tiab] OR echotomograph*[tiab])))) NOT ((Diagnosis/Narrow[filter]) AND ((("Appendix"[Mesh] OR appendix[tiab] OR appendix[ot] OR "Appendicitis"[Mesh] OR "Appendectomy"[Mesh] OR appendicit*[tiab] OR appendicit*[ot] OR appendectom*[tiab] OR appendectom*[ot] OR appendicectom*[tiab] OR appendicectom*[ot]) AND ("Diagnosis"[Mesh] OR "diagnosis"[Subheading] OR "Diagnostic Imaging"[Mesh] OR "Physical Examination"[Mesh] OR "Leukocytosis"[Mesh] OR "C-Reactive Protein"[Mesh] OR "Laparoscopy"[Mesh] OR "Magnetic Resonance Imaging"[Mesh] OR ("magnetic resonance"[tiab] AND (image[tiab] OR images[tiab] OR imaging[tiab])) OR mri[tiab] OR mris[tiab] OR nmr[tiab] OR mra[tiab] OR mras[tiab] OR zeugmatograph*[tiab] OR "mr tomography"[tiab] OR "mr tomographies"[tiab] OR "mr tomographic"[tiab] OR "proton spin"[tiab] OR ((magneti*[tiab] OR "chemical shift"[tiab]) AND imaging[tiab]) OR fmri[tiab] OR fmris[tiab] OR "Ultrasonography"[Mesh] OR "ultrasonography "[Subheading] OR ultraso*[tiab] OR sonograph*[tiab] OR echograph*[tiab] OR echocardiograph*[tiab] OR echotomograph*[tiab])))))))) OR ((("Appendix"[Mesh] OR appendix[tiab] OR appendix[ot] OR "Appendicitis"[Mesh] OR "Appendectomy"[Mesh] OR appendicit*[tiab] OR appendicit*[ot] OR appendectom*[tiab] OR appendectom*[ot] OR appendicectom*[tiab] OR appendicectom*[ot]) AND (((random*[tiab] AND (controlled[tiab] OR control[tiab] OR placebo[tiab] OR versus[tiab] OR vs[tiab] OR group[tiab] OR groups[tiab] OR comparison[tiab] OR compared[tiab] OR arm[tiab] OR arms[tiab] OR crossover[tiab] OR cross-over[tiab]) AND (trial[tiab] OR study[tiab])) OR ((single[tiab] OR double[tiab] OR triple[tiab]) AND (masked[tiab] OR blind*[tiab]))) OR ((random*[ot] AND (controlled[ot] OR control[ot] OR placebo[ot] OR versus[ot] OR vs[ot] OR group[ot] OR groups[ot] OR comparison[ot] OR compared[ot] OR arm[ot] OR arms[ot] OR crossover[ot] OR cross-over[ot]) AND (trial[ot] OR study[ot])) OR ((single[ot] OR double[ot] OR triple[ot]) AND (masked[ot] OR blind*[ot]))))) NOT (((((review*[tiab] OR search*[tiab] OR survey*[tiab] OR handsearch*[tiab] OR hand-search*[tiab]) AND (databa*[tiab] OR data-ba*[tiab] OR bibliograph*[tiab] OR electronic*[tiab] OR medline*[tiab] OR pubmed*[tiab] OR embase*[tiab] OR Cochrane[tiab] OR cinahl[tiab] OR psycinfo[tiab] OR psychinfo[tiab] OR cinhal[tiab] OR "web of science"[tiab] OR "web of knowledge"[tiab] OR ebsco[tiab] OR ovid[tiab] OR mrct[tiab] OR metaregist*[tiab] OR meta-regist*[tiab] OR ((predetermined[tiab] OR pre-determined[tiab]) AND criteri*[tiab]) OR apprais*[tiab] OR inclusion criteri*[tiab] OR exclusion criteri*[tiab]) OR (review[pt] AND systemat*[tiab]) OR "systematic review"[tiab] OR "systematic literature"[tiab] OR "integrative review"[tiab] OR "integrative literature"[tiab] OR "evidence-based review"[tiab] OR "evidence-based overview"[tiab] OR "evidence-based literature"[tiab] OR "evidence-based survey"[tiab] OR "literature search"[tiab] OR ((systemat*[ti] OR evidence-based[ti]) AND (review*[ti] OR literature[ti] OR overview[ti] OR survey[ti])) OR "data synthesis"[tiab] OR "evidence synthesis"[tiab] OR "data extraction"[tiab] OR "data source"[tiab] OR "data sources"[tiab] OR "study selection"[tiab] OR "methodological quality"[tiab] OR "methodologic quality"[tiab] OR cochrane database syst rev[ta] OR meta-analy*[tiab] OR metaanaly*[tiab] OR metanaly*[tiab] OR meta-analysis[pt] OR meta-synthesis[tiab] OR metasynthesis[tiab] OR meta-study[tiab] OR metastudy[tiab] OR metaethnograph*[tiab] OR meta-ethnograph*[tiab] OR Technology Assessment, Biomedical[mh] OR hta[tiab] OR health technol assess[ta] OR evid rep technol assess summ[ta] OR health technology assessment[tiab]) OR ((review*[ot] OR search*[ot] OR survey*[ot] OR handsearch*[ot] OR hand-search*[ot]) AND (databa*[ot] OR data-ba*[ot] OR bibliograph*[ot] OR electronic*[ot] OR medline*[ot] OR pubmed*[ot] OR embase*[ot] OR cochrane[ot] OR cinahl[ot] OR psycinfo[ot] OR psychinfo[ot] OR cinhal[ot] OR "web of science"[ot] OR "web of knowledge"[ot] OR ebsco[ot] OR ovid[ot] OR mrct[ot] OR metaregist*[ot] OR meta-regist*[ot] OR ((predetermined[ot] OR pre-determined[ot]) AND criteri*[ot]) OR apprais*[ot] OR inclusion criteri*[ot] OR exclusion criteri*[ot]) OR (review[pt] AND systemat*[ot]) OR "systematic review"[ot] OR "systematic literature"[ot] OR "integrative review"[ot] OR "integrative literature"[ot] OR "evidence-based review"[ot] OR "evidence-based overview"[ot] OR "evidence-based literature"[ot] OR "evidence-based survey"[ot] OR "literature search"[ot] OR ((systemat*[ti] OR evidence-based[ti]) AND (review*[ti] OR literature[ti] OR overview[ti] OR survey[ti])) OR "data synthesis"[ot] OR "evidence synthesis"[ot] OR "data extraction"[ot] OR "data source"[ot] OR "data sources"[ot] OR "study selection"[ot] OR "methodological quality"[ot] OR "methodologic quality"[ot] OR meta-analy*[ot] OR metaanaly*[ot] OR metanaly*[ot] OR meta-analysis[pt] OR meta-synthesis[ot] OR metasynthesis[ot] OR meta-study[ot] OR metastudy[ot] OR metaethnograph*[ot] OR meta-ethnograph*[ot] OR hta[ot] OR health technology assessment[ot])) AND ("Appendix"[Mesh] OR appendix[tiab] OR appendix[ot] OR "Appendicitis"[Mesh] OR "Appendectomy"[Mesh] OR appendicit*[tiab] OR appendicit*[ot] OR appendectom*[tiab] OR appendectom*[ot] OR appendicectom*[tiab] OR appendicectom*[ot])) OR ((Diagnosis/Narrow[filter]) AND ((("Appendix"[Mesh] OR appendix[tiab] OR appendix[ot] OR "Appendicitis"[Mesh] OR "Appendectomy"[Mesh] OR appendicit*[tiab] OR appendicit*[ot] OR appendectom*[tiab] OR appendectom*[ot] OR appendicectom*[tiab] OR appendicectom*[ot]) AND ("Diagnosis"[Mesh] OR "diagnosis"[Subheading] OR "Diagnostic Imaging"[Mesh] OR "Physical Examination"[Mesh] OR "Leukocytosis"[Mesh] OR "C-Reactive Protein"[Mesh] OR "Laparoscopy"[Mesh] OR "Magnetic Resonance Imaging"[Mesh] OR ("magnetic resonance"[tiab] AND (image[tiab] OR images[tiab] OR imaging[tiab])) OR mri[tiab] OR mris[tiab] OR nmr[tiab] OR mra[tiab] OR mras[tiab] OR zeugmatograph*[tiab] OR "mr tomography"[tiab] OR "mr tomographies"[tiab] OR "mr tomographic"[tiab] OR "proton spin"[tiab] OR ((magneti*[tiab] OR "chemical shift"[tiab]) AND imaging[tiab]) OR fmri[tiab] OR fmris[tiab] OR "Ultrasonography"[Mesh] OR "ultrasonography "[Subheading] OR ultraso*[tiab] OR sonograph*[tiab] OR echograph*[tiab] OR echocardiograph*[tiab] OR echotomograph*[tiab])))) OR (((systematic[sb] OR therapy/narrow[filter]) AND ((("Appendix"[Mesh] OR appendix[tiab] OR appendix[ot] OR "Appendicitis"[Mesh] OR "Appendectomy"[Mesh] OR appendicit*[tiab] OR appendicit*[ot] OR appendectom*[tiab] OR appendectom*[ot] OR appendicectom*[tiab] OR appendicectom*[ot]) AND ("Diagnosis"[Mesh] OR "diagnosis"[Subheading] OR "Diagnostic Imaging"[Mesh] OR "Physical Examination"[Mesh] OR "Leukocytosis"[Mesh] OR "C-Reactive Protein"[Mesh] OR "Laparoscopy"[Mesh] OR "Magnetic Resonance Imaging"[Mesh] OR ("magnetic resonance"[tiab] AND (image[tiab] OR images[tiab] OR imaging[tiab])) OR mri[tiab] OR mris[tiab] OR nmr[tiab] OR mra[tiab] OR mras[tiab] OR zeugmatograph*[tiab] OR "mr tomography"[tiab] OR "mr tomographies"[tiab] OR "mr tomographic"[tiab] OR "proton spin"[tiab] OR ((magneti*[tiab] OR "chemical shift"[tiab]) AND imaging[tiab]) OR fmri[tiab] OR fmris[tiab] OR "Ultrasonography"[Mesh] OR "ultrasonography "[Subheading] OR ultraso*[tiab] OR sonograph*[tiab] OR echograph*[tiab] OR echocardiograph*[tiab] OR echotomograph*[tiab])))) NOT ((Diagnosis/Narrow[filter]) AND ((("Appendix"[Mesh] OR appendix[tiab] OR appendix[ot] OR "Appendicitis"[Mesh] OR "Appendectomy"[Mesh] OR appendicit*[tiab] OR appendicit*[ot] OR appendectom*[tiab] OR appendectom*[ot] OR appendicectom*[tiab] OR appendicectom*[ot]) AND ("Diagnosis"[Mesh] OR "diagnosis"[Subheading] OR "Diagnostic Imaging"[Mesh] OR "Physical Examination"[Mesh] OR "Leukocytosis"[Mesh] OR "C-Reactive Protein"[Mesh] OR "Laparoscopy"[Mesh] OR "Magnetic Resonance Imaging"[Mesh] OR ("magnetic resonance"[tiab] AND (image[tiab] OR images[tiab] OR imaging[tiab])) OR mri[tiab] OR mris[tiab] OR nmr[tiab] OR mra[tiab] OR mras[tiab] OR zeugmatograph*[tiab] OR "mr tomography"[tiab] OR "mr tomographies"[tiab] OR "mr tomographic"[tiab] OR "proton spin"[tiab] OR ((magneti*[tiab] OR "chemical shift"[tiab]) AND imaging[tiab]) OR fmri[tiab] OR fmris[tiab] OR "Ultrasonography"[Mesh] OR "ultrasonography "[Subheading] OR ultraso*[tiab] OR sonograph*[tiab] OR echograph*[tiab] OR echocardiograph*[tiab] OR echotomograph*[tiab])))))))) OR (((((("Incidence"[Majr]) OR "Prevalence"[Majr]) OR incidence*[ti] OR prevalen*[ti] OR incidence*[ot] OR prevalen*[ot]))) AND ("Appendix"[Mesh] OR appendix[tiab] OR appendix[ot] OR "Appendicitis"[Mesh] OR appendicit*[tiab] OR appendicit*[ot]))) OR (("Appendix"[Mesh] OR appendix[tiab] OR appendix[ot] OR "Appendicitis"[Mesh] OR "Appendectomy"[Mesh] OR appendicit*[tiab] OR appendicit*[ot] OR appendectom*[tiab] OR appendectom*[ot] OR appendicectom*[tiab] OR appendicectom*[ot]) AND ("Diagnosis"[Mesh] OR "diagnosis"[Subheading] OR "Diagnostic Imaging"[Mesh] OR "Physical Examination"[Mesh] OR "Leukocytosis"[Mesh] OR "C-Reactive Protein"[Mesh] OR "Laparoscopy"[Mesh] OR "Magnetic Resonance Imaging"[Mesh] OR "Ultrasonography"[Mesh] OR "ultrasonography "[Subheading] OR c reactive protein*[tiab] OR crp[tiab] OR diagnos*[tiab] OR examinati*[tiab] OR palpati*[tiab] OR leukocytos*[tiab] OR pleocytos*[tiab] OR laparoscop*[tiab] OR ("magnetic resonance"[tiab] AND (image[tiab] OR images[tiab] OR imaging[tiab])) OR mri[tiab] OR mris[tiab] OR nmr[tiab] OR mra[tiab] OR mras[tiab] OR zeugmatograph*[tiab] OR mr tomograph*[tiab] OR "proton spin"[tiab] OR ((magneti*[tiab] OR "chemical shift"[tiab]) AND imaging[tiab]) OR fmri[tiab] OR fmris[tiab] OR ultraso*[tiab] OR sonograph*[tiab] OR echograph*[tiab] OR echocardiograph*[tiab] OR echotomograph*[tiab] OR imaging*[tiab] OR c reactive protein*[ot] OR crp[ot] OR diagnos*[ot] OR examinati*[ot] OR palpati*[ot] OR leukocytos*[ot] OR pleocytos*[ot] OR laparoscop*[ot] OR ("magnetic resonance"[ot] AND (image[ot] OR images[ot] OR imaging[ot])) OR mri[ot] OR mris[ot] OR nmr[ot] OR mra[ot] OR mras[ot] OR zeugmatograph*[ot] OR mr tomograph*[ot] OR "proton spin"[ot] OR ((magneti*[ot] OR "chemical shift"[ot]) AND imaging[ot]) OR fmri[ot] OR fmris[ot] OR ultraso*[ot] OR sonograph*[ot] OR echograph*[ot] OR echocardiograph*[ot] OR echotomograph*[ot] OR imaging*[ot]) AND (((review*[tiab] OR search*[tiab] OR survey*[tiab] OR handsearch*[tiab] OR hand-search*[tiab]) AND (databa*[tiab] OR data-ba*[tiab] OR bibliograph*[tiab] OR electronic*[tiab] OR medline*[tiab] OR pubmed*[tiab] OR embase*[tiab] OR Cochrane[tiab] OR cinahl[tiab] OR psycinfo[tiab] OR psychinfo[tiab] OR cinhal[tiab] OR "web of science"[tiab] OR "web of knowledge"[tiab] OR ebsco[tiab] OR ovid[tiab] OR mrct[tiab] OR metaregist*[tiab] OR meta-regist*[tiab] OR ((predetermined[tiab] OR pre-determined[tiab]) AND criteri*[tiab]) OR apprais*[tiab] OR inclusion criteri*[tiab] OR exclusion criteri*[tiab]) OR (review[pt] AND systemat*[tiab]) OR "systematic review"[tiab] OR "systematic literature"[tiab] OR "integrative review"[tiab] OR "integrative literature"[tiab] OR "evidence-based review"[tiab] OR "evidence-based overview"[tiab] OR "evidence-based literature"[tiab] OR "evidence-based survey"[tiab] OR "literature search"[tiab] OR ((systemat*[ti] OR evidence-based[ti]) AND (review*[ti] OR literature[ti] OR overview[ti] OR survey[ti])) OR "data synthesis"[tiab] OR "evidence synthesis"[tiab] OR "data extraction"[tiab] OR "data source"[tiab] OR "data sources"[tiab] OR "study selection"[tiab] OR "methodological quality"[tiab] OR "methodologic quality"[tiab] OR cochrane database syst rev[ta] OR meta-analy*[tiab] OR metaanaly*[tiab] OR metanaly*[tiab] OR meta-analysis[pt] OR meta-synthesis[tiab] OR metasynthesis[tiab] OR meta-study[tiab] OR metastudy[tiab] OR metaethnograph*[tiab] OR meta-ethnograph*[tiab] OR Technology Assessment, Biomedical[mh] OR hta[tiab] OR health technol assess[ta] OR evid rep technol assess summ[ta] OR health technology assessment[tiab]) OR ((review*[ot] OR search*[ot] OR survey*[ot] OR handsearch*[ot] OR hand-search*[ot]) AND (databa*[ot] OR data-ba*[ot] OR bibliograph*[ot] OR electronic*[ot] OR medline*[ot] OR pubmed*[ot] OR embase*[ot] OR cochrane[ot] OR cinahl[ot] OR psycinfo[ot] OR psychinfo[ot] OR cinhal[ot] OR "web of science"[ot] OR "web of knowledge"[ot] OR ebsco[ot] OR ovid[ot] OR mrct[ot] OR metaregist*[ot] OR meta-regist*[ot] OR ((predetermined[ot] OR pre-determined[ot]) AND criteri*[ot]) OR apprais*[ot] OR inclusion criteri*[ot] OR exclusion criteri*[ot]) OR (review[pt] AND systemat*[ot]) OR "systematic review"[ot] OR "systematic literature"[ot] OR "integrative review"[ot] OR "integrative literature"[ot] OR "evidence-based review"[ot] OR "evidence-based overview"[ot] OR "evidence-based literature"[ot] OR "evidence-based survey"[ot] OR "literature search"[ot] OR ((systemat*[ti] OR evidence-based[ti]) AND (review*[ti] OR literature[ti] OR overview[ti] OR survey[ti])) OR "data synthesis"[ot] OR "evidence synthesis"[ot] OR "data extraction"[ot] OR "data source"[ot] OR "data sources"[ot] OR "study selection"[ot] OR "methodological quality"[ot] OR "methodologic quality"[ot] OR meta-analy*[ot] OR metaanaly*[ot] OR metanaly*[ot] OR meta-analysis[pt] OR meta-synthesis[ot] OR metasynthesis[ot] OR meta-study[ot] OR metastudy[ot] OR metaethnograph*[ot] OR meta-ethnograph*[ot] OR hta[ot] OR health technology assessment[ot])))) OR ((("Appendix"[Mesh] OR appendix[tiab] OR appendix[ot] OR "Appendicitis"[Mesh] OR "Appendectomy"[Mesh] OR appendicit*[tiab] OR appendicit*[ot] OR appendectom*[tiab] OR appendectom*[ot] OR appendicectom*[tiab] OR appendicectom*[ot]) AND ("Diagnosis"[Mesh] OR "diagnosis"[Subheading] OR "Diagnostic Imaging"[Mesh] OR "Physical Examination"[Mesh] OR "Leukocytosis"[Mesh] OR "C-Reactive Protein"[Mesh] OR "Laparoscopy"[Mesh] OR "Magnetic Resonance Imaging"[Mesh] OR "Ultrasonography"[Mesh] OR "ultrasonography "[Subheading] OR c reactive protein*[tiab] OR crp[tiab] OR diagnos*[tiab] OR examinati*[tiab] OR palpati*[tiab] OR leukocytos*[tiab] OR pleocytos*[tiab] OR laparoscop*[tiab] OR ("magnetic resonance"[tiab] AND (image[tiab] OR images[tiab] OR imaging[tiab])) OR mri[tiab] OR mris[tiab] OR nmr[tiab] OR mra[tiab] OR mras[tiab] OR zeugmatograph*[tiab] OR mr tomograph*[tiab] OR "proton spin"[tiab] OR ((magneti*[tiab] OR "chemical shift"[tiab]) AND imaging[tiab]) OR fmri[tiab] OR fmris[tiab] OR ultraso*[tiab] OR sonograph*[tiab] OR echograph*[tiab] OR echocardiograph*[tiab] OR echotomograph*[tiab] OR imaging*[tiab] OR c reactive protein*[ot] OR crp[ot] OR diagnos*[ot] OR examinati*[ot] OR palpati*[ot] OR leukocytos*[ot] OR pleocytos*[ot] OR laparoscop*[ot] OR ("magnetic resonance"[ot] AND (image[ot] OR images[ot] OR imaging[ot])) OR mri[ot] OR mris[ot] OR nmr[ot] OR mra[ot] OR mras[ot] OR zeugmatograph*[ot] OR mr tomograph*[ot] OR "proton spin"[ot] OR ((magneti*[ot] OR "chemical shift"[ot]) AND imaging[ot]) OR fmri[ot] OR fmris[ot] OR ultraso*[ot] OR sonograph*[ot] OR echograph*[ot] OR echocardiograph*[ot] OR echotomograph*[ot] OR imaging*[ot]) AND (sensitivity and specificity[Mesh] OR sensitiv*[tiab] OR specificity[tiab] OR sensitiv*[ot] OR specificity[ot])) NOT (("Appendix"[Mesh] OR appendix[tiab] OR appendix[ot] OR "Appendicitis"[Mesh] OR "Appendectomy"[Mesh] OR appendicit*[tiab] OR appendicit*[ot] OR appendectom*[tiab] OR appendectom*[ot] OR appendicectom*[tiab] OR appendicectom*[ot]) AND ("Diagnosis"[Mesh] OR "diagnosis"[Subheading] OR "Diagnostic Imaging"[Mesh] OR "Physical Examination"[Mesh] OR "Leukocytosis"[Mesh] OR "C-Reactive Protein"[Mesh] OR "Laparoscopy"[Mesh] OR "Magnetic Resonance Imaging"[Mesh] OR "Ultrasonography"[Mesh] OR "ultrasonography "[Subheading] OR c reactive protein*[tiab] OR crp[tiab] OR diagnos*[tiab] OR examinati*[tiab] OR palpati*[tiab] OR leukocytos*[tiab] OR pleocytos*[tiab] OR laparoscop*[tiab] OR ("magnetic resonance"[tiab] AND (image[tiab] OR images[tiab] OR imaging[tiab])) OR mri[tiab] OR mris[tiab] OR nmr[tiab] OR mra[tiab] OR mras[tiab] OR zeugmatograph*[tiab] OR mr tomograph*[tiab] OR "proton spin"[tiab] OR ((magneti*[tiab] OR "chemical shift"[tiab]) AND imaging[tiab]) OR fmri[tiab] OR fmris[tiab] OR ultraso*[tiab] OR sonograph*[tiab] OR echograph*[tiab] OR echocardiograph*[tiab] OR echotomograph*[tiab] OR imaging*[tiab] OR c reactive protein*[ot] OR crp[ot] OR diagnos*[ot] OR examinati*[ot] OR palpati*[ot] OR leukocytos*[ot] OR pleocytos*[ot] OR laparoscop*[ot] OR ("magnetic resonance"[ot] AND (image[ot] OR images[ot] OR imaging[ot])) OR mri[ot] OR mris[ot] OR nmr[ot] OR mra[ot] OR mras[ot] OR zeugmatograph*[ot] OR mr tomograph*[ot] OR "proton spin"[ot] OR ((magneti*[ot] OR "chemical shift"[ot]) AND imaging[ot]) OR fmri[ot] OR fmris[ot] OR ultraso*[ot] OR sonograph*[ot] OR echograph*[ot] OR echocardiograph*[ot] OR echotomograph*[ot] OR imaging*[ot]) AND (((review*[tiab] OR search*[tiab] OR survey*[tiab] OR handsearch*[tiab] OR hand-search*[tiab]) AND (databa*[tiab] OR data-ba*[tiab] OR bibliograph*[tiab] OR electronic*[tiab] OR medline*[tiab] OR pubmed*[tiab] OR embase*[tiab] OR Cochrane[tiab] OR cinahl[tiab] OR psycinfo[tiab] OR psychinfo[tiab] OR cinhal[tiab] OR "web of science"[tiab] OR "web of knowledge"[tiab] OR ebsco[tiab] OR ovid[tiab] OR mrct[tiab] OR metaregist*[tiab] OR meta-regist*[tiab] OR ((predetermined[tiab] OR pre-determined[tiab]) AND criteri*[tiab]) OR apprais*[tiab] OR inclusion criteri*[tiab] OR exclusion criteri*[tiab]) OR (review[pt] AND systemat*[tiab]) OR "systematic review"[tiab] OR "systematic literature"[tiab] OR "integrative review"[tiab] OR "integrative literature"[tiab] OR "evidence-based review"[tiab] OR "evidence-based overview"[tiab] OR "evidence-based literature"[tiab] OR "evidence-based survey"[tiab] OR "literature search"[tiab] OR ((systemat*[ti] OR evidence-based[ti]) AND (review*[ti] OR literature[ti] OR overview[ti] OR survey[ti])) OR "data synthesis"[tiab] OR "evidence synthesis"[tiab] OR "data extraction"[tiab] OR "data source"[tiab] OR "data sources"[tiab] OR "study selection"[tiab] OR "methodological quality"[tiab] OR "methodologic quality"[tiab] OR cochrane database syst rev[ta] OR meta-analy*[tiab] OR metaanaly*[tiab] OR metanaly*[tiab] OR meta-analysis[pt] OR meta-synthesis[tiab] OR metasynthesis[tiab] OR meta-study[tiab] OR metastudy[tiab] OR metaethnograph*[tiab] OR meta-ethnograph*[tiab] OR Technology Assessment, Biomedical[mh] OR hta[tiab] OR health technol assess[ta] OR evid rep technol assess summ[ta] OR health technology assessment[tiab]) OR ((review*[ot] OR search*[ot] OR survey*[ot] OR handsearch*[ot] OR hand-search*[ot]) AND (databa*[ot] OR data-ba*[ot] OR bibliograph*[ot] OR electronic*[ot] OR medline*[ot] OR pubmed*[ot] OR embase*[ot] OR cochrane[ot] OR cinahl[ot] OR psycinfo[ot] OR psychinfo[ot] OR cinhal[ot] OR "web of science"[ot] OR "web of knowledge"[ot] OR ebsco[ot] OR ovid[ot] OR mrct[ot] OR metaregist*[ot] OR meta-regist*[ot] OR ((predetermined[ot] OR pre-determined[ot]) AND criteri*[ot]) OR apprais*[ot] OR inclusion criteri*[ot] OR exclusion criteri*[ot]) OR (review[pt] AND systemat*[ot]) OR "systematic review"[ot] OR "systematic literature"[ot] OR "integrative review"[ot] OR "integrative literature"[ot] OR "evidence-based review"[ot] OR "evidence-based overview"[ot] OR "evidence-based literature"[ot] OR "evidence-based survey"[ot] OR "literature search"[ot] OR ((systemat*[ti] OR evidence-based[ti]) AND (review*[ti] OR literature[ti] OR overview[ti] OR survey[ti])) OR "data synthesis"[ot] OR "evidence synthesis"[ot] OR "data extraction"[ot] OR "data source"[ot] OR "data sources"[ot] OR "study selection"[ot] OR "methodological quality"[ot] OR "methodologic quality"[ot] OR meta-analy*[ot] OR metaanaly*[ot] OR metanaly*[ot] OR meta-analysis[pt] OR meta-synthesis[ot] OR metasynthesis[ot] OR meta-study[ot] OR metastudy[ot] OR metaethnograph*[ot] OR meta-ethnograph*[ot] OR hta[ot] OR health technology assessment[ot]))))) OR ((("Appendix"[Mesh] OR appendix[tiab] OR appendix[ot] OR "Appendicitis"[Mesh] OR "Appendectomy"[Mesh] OR appendicit*[tiab] OR appendicit*[ot] OR appendectom*[tiab] OR appendectom*[ot] OR appendicectom*[tiab] OR appendicectom*[ot]) AND (("Learning Curve"[Mesh] OR learning curve*[tiab] OR skill*[tiab] OR expertise*[tiab]) OR "Professional Competence"[Mesh] OR competenc*[tiab]) AND ((therapy/narrow[filter]) OR (((review*[tiab] OR search*[tiab] OR survey*[tiab] OR handsearch*[tiab] OR hand-search*[tiab]) AND (databa*[tiab] OR data-ba*[tiab] OR bibliograph*[tiab] OR electronic*[tiab] OR medline*[tiab] OR pubmed*[tiab] OR embase*[tiab] OR Cochrane[tiab] OR cinahl[tiab] OR psycinfo[tiab] OR psychinfo[tiab] OR cinhal[tiab] OR "web of science"[tiab] OR "web of knowledge"[tiab] OR ebsco[tiab] OR ovid[tiab] OR mrct[tiab] OR metaregist*[tiab] OR meta-regist*[tiab] OR ((predetermined[tiab] OR pre-determined[tiab]) AND criteri*[tiab]) OR apprais*[tiab] OR inclusion criteri*[tiab] OR exclusion criteri*[tiab]) OR (review[pt] AND systemat*[tiab]) OR "systematic review"[tiab] OR "systematic literature"[tiab] OR "integrative review"[tiab] OR "integrative literature"[tiab] OR "evidence-based review"[tiab] OR "evidence-based overview"[tiab] OR "evidence-based literature"[tiab] OR "evidence-based survey"[tiab] OR "literature search"[tiab] OR ((systemat*[ti] OR evidence-based[ti]) AND (review*[ti] OR literature[ti] OR overview[ti] OR survey[ti])) OR "data synthesis"[tiab] OR "evidence synthesis"[tiab] OR "data extraction"[tiab] OR "data source"[tiab] OR "data sources"[tiab] OR "study selection"[tiab] OR "methodological quality"[tiab] OR "methodologic quality"[tiab] OR cochrane database syst rev[ta] OR meta-analy*[tiab] OR metaanaly*[tiab] OR metanaly*[tiab] OR meta-analysis[pt] OR meta-synthesis[tiab] OR metasynthesis[tiab] OR meta-study[tiab] OR metastudy[tiab] OR metaethnograph*[tiab] OR meta-ethnograph*[tiab] OR Technology Assessment, Biomedical[mh] OR hta[tiab] OR health technol assess[ta] OR evid rep technol assess summ[ta] OR health technology assessment[tiab]) OR ((review*[ot] OR search*[ot] OR survey*[ot] OR handsearch*[ot] OR hand-search*[ot]) AND (databa*[ot] OR data-ba*[ot] OR bibliograph*[ot] OR electronic*[ot] OR medline*[ot] OR pubmed*[ot] OR embase*[ot] OR cochrane[ot] OR cinahl[ot] OR psycinfo[ot] OR psychinfo[ot] OR cinhal[ot] OR "web of science"[ot] OR "web of knowledge"[ot] OR ebsco[ot] OR ovid[ot] OR mrct[ot] OR metaregist*[ot] OR meta-regist*[ot] OR ((predetermined[ot] OR pre-determined[ot]) AND criteri*[ot]) OR apprais*[ot] OR inclusion criteri*[ot] OR exclusion criteri*[ot]) OR (review[pt] AND systemat*[ot]) OR "systematic review"[ot] OR "systematic literature"[ot] OR "integrative review"[ot] OR "integrative literature"[ot] OR "evidence-based review"[ot] OR "evidence-based overview"[ot] OR "evidence-based literature"[ot] OR "evidence-based survey"[ot] OR "literature search"[ot] OR ((systemat*[ti] OR evidence-based[ti]) AND (review*[ti] OR literature[ti] OR overview[ti] OR survey[ti])) OR "data synthesis"[ot] OR "evidence synthesis"[ot] OR "data extraction"[ot] OR "data source"[ot] OR "data sources"[ot] OR "study selection"[ot] OR "methodological quality"[ot] OR "methodologic quality"[ot] OR meta-analy*[ot] OR metaanaly*[ot] OR metanaly*[ot] OR meta-analysis[pt] OR meta-synthesis[ot] OR metasynthesis[ot] OR meta-study[ot] OR metastudy[ot] OR metaethnograph*[ot] OR meta-ethnograph*[ot] OR hta[ot] OR health technology assessment[ot])))) OR (("Appendix"[Mesh] OR appendix[tiab] OR appendix[ot] OR "Appendicitis"[Mesh] OR "Appendectomy"[Mesh] OR appendicit*[tiab] OR appendicit*[ot] OR appendectom*[tiab] OR appendectom*[ot] OR appendicectom*[tiab] OR appendicectom*[ot]) AND (("Learning Curve"[Majr] OR learning curve*[ti] OR skill*[ti] OR expertise*[ti]) OR "Professional Competence"[Majr] OR competenc*[ti])))) OR ((("Appendix"[Mesh] OR appendix[tiab] OR appendix[ot] OR "Appendicitis"[Mesh] OR "Appendectomy"[Mesh] OR appendicit*[tiab] OR appendicit*[ot] OR appendectom*[tiab] OR appendectom*[ot] OR appendicectom*[tiab] OR appendicectom*[ot]) AND ("Antibiotic Prophylaxis"[Mesh] OR ((antibiotic*[tiab] OR antibacteri*[tiab]) AND (prophylax*[tiab] OR premedicati*[tiab])) OR "Intubation, Gastrointestinal"[Mesh] OR ((Nasogastric[tiab] OR gastric[tiab]) AND (intubation*[tiab] OR tube[tiab] OR tubes[tiab])) OR "Urinary Catheters"[Mesh] OR "Urinary Catheterization"[Mesh] OR (urin*[tiab] AND catheter*[tiab]) OR "Patient Positioning"[Mesh] OR ((patient*[tiab] OR table*[tiab] OR "Operating Tables"[Mesh]) AND position*[tiab]) OR "Pneumoperitoneum, Artificial"[Mesh] OR pneumoperiton*[tiab] OR veress*[tiab] OR trocar*[tiab] OR open introduc*[tiab]) AND ((therapy/narrow[filter]) OR (((review*[tiab] OR search*[tiab] OR survey*[tiab] OR handsearch*[tiab] OR hand-search*[tiab]) AND (databa*[tiab] OR data-ba*[tiab] OR bibliograph*[tiab] OR electronic*[tiab] OR medline*[tiab] OR pubmed*[tiab] OR embase*[tiab] OR Cochrane[tiab] OR cinahl[tiab] OR psycinfo[tiab] OR psychinfo[tiab] OR cinhal[tiab] OR "web of science"[tiab] OR "web of knowledge"[tiab] OR ebsco[tiab] OR ovid[tiab] OR mrct[tiab] OR metaregist*[tiab] OR meta-regist*[tiab] OR ((predetermined[tiab] OR pre-determined[tiab]) AND criteri*[tiab]) OR apprais*[tiab] OR inclusion criteri*[tiab] OR exclusion criteri*[tiab]) OR (review[pt] AND systemat*[tiab]) OR "systematic review"[tiab] OR "systematic literature"[tiab] OR "integrative review"[tiab] OR "integrative literature"[tiab] OR "evidence-based review"[tiab] OR "evidence-based overview"[tiab] OR "evidence-based literature"[tiab] OR "evidence-based survey"[tiab] OR "literature search"[tiab] OR ((systemat*[ti] OR evidence-based[ti]) AND (review*[ti] OR literature[ti] OR overview[ti] OR survey[ti])) OR "data synthesis"[tiab] OR "evidence synthesis"[tiab] OR "data extraction"[tiab] OR "data source"[tiab] OR "data sources"[tiab] OR "study selection"[tiab] OR "methodological quality"[tiab] OR "methodologic quality"[tiab] OR cochrane database syst rev[ta] OR meta-analy*[tiab] OR metaanaly*[tiab] OR metanaly*[tiab] OR meta-analysis[pt] OR meta-synthesis[tiab] OR metasynthesis[tiab] OR meta-study[tiab] OR metastudy[tiab] OR metaethnograph*[tiab] OR meta-ethnograph*[tiab] OR Technology Assessment, Biomedical[mh] OR hta[tiab] OR health technol assess[ta] OR evid rep technol assess summ[ta] OR health technology assessment[tiab]) OR ((review*[ot] OR search*[ot] OR survey*[ot] OR handsearch*[ot] OR hand-search*[ot]) AND (databa*[ot] OR data-ba*[ot] OR bibliograph*[ot] OR electronic*[ot] OR medline*[ot] OR pubmed*[ot] OR embase*[ot] OR cochrane[ot] OR cinahl[ot] OR psycinfo[ot] OR psychinfo[ot] OR cinhal[ot] OR "web of science"[ot] OR "web of knowledge"[ot] OR ebsco[ot] OR ovid[ot] OR mrct[ot] OR metaregist*[ot] OR meta-regist*[ot] OR ((predetermined[ot] OR pre-determined[ot]) AND criteri*[ot]) OR apprais*[ot] OR inclusion criteri*[ot] OR exclusion criteri*[ot]) OR (review[pt] AND systemat*[ot]) OR "systematic review"[ot] OR "systematic literature"[ot] OR "integrative review"[ot] OR "integrative literature"[ot] OR "evidence-based review"[ot] OR "evidence-based overview"[ot] OR "evidence-based literature"[ot] OR "evidence-based survey"[ot] OR "literature search"[ot] OR ((systemat*[ti] OR evidence-based[ti]) AND (review*[ti] OR literature[ti] OR overview[ti] OR survey[ti])) OR "data synthesis"[ot] OR "evidence synthesis"[ot] OR "data extraction"[ot] OR "data source"[ot] OR "data sources"[ot] OR "study selection"[ot] OR "methodological quality"[ot] OR "methodologic quality"[ot] OR meta-analy*[ot] OR metaanaly*[ot] OR metanaly*[ot] OR meta-analysis[pt] OR meta-synthesis[ot] OR metasynthesis[ot] OR meta-study[ot] OR metastudy[ot] OR metaethnograph*[ot] OR meta-ethnograph*[ot] OR hta[ot] OR health technology assessment[ot])))) OR (("Appendix"[Mesh] OR appendix[tiab] OR appendix[ot] OR "Appendicitis"[Mesh] OR "Appendectomy"[Mesh] OR appendicit*[tiab] OR appendicit*[ot] OR appendectom*[tiab] OR appendectom*[ot] OR appendicectom*[tiab] OR appendicectom*[ot]) AND ("Antibiotic Prophylaxis"[Majr] OR ((antibiotic*[ti] OR antibacteri*[ti]) AND (prophylax*[ti] OR premedicati*[ti])) OR "Intubation, Gastrointestinal"[Majr] OR ((Nasogastric[ti] OR gastric[ti]) AND (intubation*[ti] OR tube[ti] OR tubes[ti])) OR "Urinary Catheters"[Majr] OR "Urinary Catheterization"[Majr] OR (urin*[ti] AND catheter*[ti]) OR "Patient Positioning"[Majr] OR ((patient*[ti] OR table*[ti] OR "Operating Tables"[Majr]) AND position*[ti]) OR "Pneumoperitoneum, Artificial"[Majr] OR pneumoperiton*[ti] OR veress*[ti] OR trocar*[ti] OR open introduc*[ti])))) OR (((("Appendix"[Mesh] OR appendix[tiab] OR appendix[ot] OR "Appendicitis"[Mesh] OR "Appendectomy"[Mesh] OR appendicit*[tiab] OR appendicit*[ot] OR appendectom*[tiab] OR appendectom*[ot] OR appendicectom*[tiab] OR appendicectom*[ot]) AND (appendix sana[tiab] OR "Surgical Instruments"[Mesh] OR clip*[tiab] OR stapl*[tiab] OR endoclip*[tiab] OR endostapl*[tiab] OR loop[tiab] OR loops[tiab] OR endoloop*[tiab] OR skeletiz*[tiab] OR skeletis*[tiab] OR "Diathermy"[Mesh] OR diatherm*[tiab] OR "Sutures"[Mesh] OR sutur*[tiab] OR endobag*[tiab] OR glove*[tiab] OR "Gloves, Surgical"[Mesh] OR trocar*[tiab] OR extracti*[tiab] OR ((intraabdom*[tiab] OR abdom*[tiab]) AND fluid*[tiab] OR purulen*[tiab]) OR "Suppuration"[Mesh] OR pus[tiab] OR suppurati*[tiab] OR abscess*[tiab] OR "Conversion to Open Surgery"[Mesh] OR conver*[tiab] OR culture*[tiab] OR swab*[tiab]) AND ((((review*[tiab] OR search*[tiab] OR survey*[tiab] OR handsearch*[tiab] OR hand-search*[tiab]) AND (databa*[tiab] OR data-ba*[tiab] OR bibliograph*[tiab] OR electronic*[tiab] OR medline*[tiab] OR pubmed*[tiab] OR embase*[tiab] OR Cochrane[tiab] OR cinahl[tiab] OR psycinfo[tiab] OR psychinfo[tiab] OR cinhal[tiab] OR "web of science"[tiab] OR "web of knowledge"[tiab] OR ebsco[tiab] OR ovid[tiab] OR mrct[tiab] OR metaregist*[tiab] OR meta-regist*[tiab] OR ((predetermined[tiab] OR pre-determined[tiab]) AND criteri*[tiab]) OR apprais*[tiab] OR inclusion criteri*[tiab] OR exclusion criteri*[tiab]) OR (review[pt] AND systemat*[tiab]) OR "systematic review"[tiab] OR "systematic literature"[tiab] OR "integrative review"[tiab] OR "integrative literature"[tiab] OR "evidence-based review"[tiab] OR "evidence-based overview"[tiab] OR "evidence-based literature"[tiab] OR "evidence-based survey"[tiab] OR "literature search"[tiab] OR ((systemat*[ti] OR evidence-based[ti]) AND (review*[ti] OR literature[ti] OR overview[ti] OR survey[ti])) OR "data synthesis"[tiab] OR "evidence synthesis"[tiab] OR "data extraction"[tiab] OR "data source"[tiab] OR "data sources"[tiab] OR "study selection"[tiab] OR "methodological quality"[tiab] OR "methodologic quality"[tiab] OR cochrane database syst rev[ta] OR meta-analy*[tiab] OR metaanaly*[tiab] OR metanaly*[tiab] OR meta-analysis[pt] OR meta-synthesis[tiab] OR metasynthesis[tiab] OR meta-study[tiab] OR metastudy[tiab] OR metaethnograph*[tiab] OR meta-ethnograph*[tiab] OR Technology Assessment, Biomedical[mh] OR hta[tiab] OR health technol assess[ta] OR evid rep technol assess summ[ta] OR health technology assessment[tiab]) OR ((review*[ot] OR search*[ot] OR survey*[ot] OR handsearch*[ot] OR hand-search*[ot]) AND (databa*[ot] OR data-ba*[ot] OR bibliograph*[ot] OR electronic*[ot] OR medline*[ot] OR pubmed*[ot] OR embase*[ot] OR cochrane[ot] OR cinahl[ot] OR psycinfo[ot] OR psychinfo[ot] OR cinhal[ot] OR "web of science"[ot] OR "web of knowledge"[ot] OR ebsco[ot] OR ovid[ot] OR mrct[ot] OR metaregist*[ot] OR meta-regist*[ot] OR ((predetermined[ot] OR pre-determined[ot]) AND criteri*[ot]) OR apprais*[ot] OR inclusion criteri*[ot] OR exclusion criteri*[ot]) OR (review[pt] AND systemat*[ot]) OR "systematic review"[ot] OR "systematic literature"[ot] OR "integrative review"[ot] OR "integrative literature"[ot] OR "evidence-based review"[ot] OR "evidence-based overview"[ot] OR "evidence-based literature"[ot] OR "evidence-based survey"[ot] OR "literature search"[ot] OR ((systemat*[ti] OR evidence-based[ti]) AND (review*[ti] OR literature[ti] OR overview[ti] OR survey[ti])) OR "data synthesis"[ot] OR "evidence synthesis"[ot] OR "data extraction"[ot] OR "data source"[ot] OR "data sources"[ot] OR "study selection"[ot] OR "methodological quality"[ot] OR "methodologic quality"[ot] OR meta-analy*[ot] OR metaanaly*[ot] OR metanaly*[ot] OR meta-analysis[pt] OR meta-synthesis[ot] OR metasynthesis[ot] OR meta-study[ot] OR metastudy[ot] OR metaethnograph*[ot] OR meta-ethnograph*[ot] OR hta[ot] OR health technology assessment[ot])) OR (therapy/narrow[filter])))) OR (("Appendix"[Mesh] OR appendix[tiab] OR appendix[ot] OR "Appendicitis"[Mesh] OR "Appendectomy"[Mesh] OR appendicit*[tiab] OR appendicit*[ot] OR appendectom*[tiab] OR appendectom*[ot] OR appendicectom*[tiab] OR appendicectom*[ot]) AND (appendix sana[ti] OR "Surgical Instruments"[majr] OR clip*[ti] OR stapl*[ti] OR endoclip*[ti] OR endostapl*[ti] OR loop[ti] OR loops[ti] OR endoloop*[ti] OR skeletiz*[ti] OR skeletis*[ti] OR "Diathermy"[majr] OR diatherm*[ti] OR "Sutures"[majr] OR sutur*[ti] OR endobag*[ti] OR glove*[ti] OR "Gloves, Surgical"[majr] OR trocar*[ti] OR extracti*[ti] OR ((intraabdom*[ti] OR abdom*[ti]) AND fluid*[ti] OR purulen*[ti]) OR "Suppuration"[majr] OR pus[ti] OR suppurati*[ti] OR abscess*[ti] OR "Conversion to Open Surgery"[majr] OR conver*[ti] OR culture*[ti] OR swab*[ti])))) OR (("Postoperative Care"[Mesh] OR "Postoperative Complications"[Mesh] OR "Postoperative Period"[Mesh] OR "Recurrence"[Mesh] OR "Patient Discharge"[Mesh] OR "Cicatrix"[Mesh] OR "Tissue Adhesions"[Mesh] OR "Intestinal Obstruction"[Mesh] OR "Infertility"[Mesh] OR "Iatrogenic Disease"[Mesh] OR postoperati*[tiab] OR wound infecti*[tiab] OR swi[tiab] OR swis[tiab] OR ssi[tiab] OR ssis[tiab] OR surgical site infecti*[tiab] OR dehiscen*[tiab] OR leak*[tiab] OR discharg*[tiab] OR fast track*[tiab] OR fasttrack*[tiab] OR scar[tiab] OR scars[tiab] OR scarring[tiab] OR cicatri*[tiab] OR stump*[tiab] OR incision*[tiab] OR adhesion*[tiab] OR Intestinal Obstruction*[tiab] OR ileus[tiab] OR infertil*[tiab] OR subfertil*[tiab] OR iatrogen*[tiab] OR postoperati*[ot] OR recurren*[ot] OR relaps*[ot] OR recrudescenc*[ot] OR wound infecti*[ot] OR swi[ot] OR swis[ot] OR ssi[ot] OR ssis[ot] OR surgical site infecti*[ot] OR dehiscen*[ot] OR leak*[ot] OR discharg*[ot] OR fast track*[ot] OR fasttrack*[ot] OR scar[ot] OR scars[ot] OR scarring[ot] OR cicatri*[ot] OR stump*[ot] OR incision*[ot] OR adhesion*[ot] OR Intestinal Obstruction*[ot] OR ileus[ot] OR infertil*[ot] OR subfertil*[ot] OR iatrogen*[ot]) AND ("Appendix"[Mesh] OR appendix[tiab] OR appendix[ot] OR "Appendicitis"[Mesh] OR "Appendectomy"[Mesh] OR appendicit*[tiab] OR appendicit*[ot] OR appendectom*[tiab] OR appendectom*[ot] OR appendicectom*[tiab] OR appendicectom*[ot]) AND (((review*[tiab] OR search*[tiab] OR survey*[tiab] OR handsearch*[tiab] OR hand-search*[tiab]) AND (databa*[tiab] OR data-ba*[tiab] OR bibliograph*[tiab] OR electronic*[tiab] OR medline*[tiab] OR pubmed*[tiab] OR embase*[tiab] OR Cochrane[tiab] OR cinahl[tiab] OR psycinfo[tiab] OR psychinfo[tiab] OR cinhal[tiab] OR "web of science"[tiab] OR "web of knowledge"[tiab] OR ebsco[tiab] OR ovid[tiab] OR mrct[tiab] OR metaregist*[tiab] OR meta-regist*[tiab] OR ((predetermined[tiab] OR pre-determined[tiab]) AND criteri*[tiab]) OR apprais*[tiab] OR inclusion criteri*[tiab] OR exclusion criteri*[tiab]) OR (review[pt] AND systemat*[tiab]) OR "systematic review"[tiab] OR "systematic literature"[tiab] OR "integrative review"[tiab] OR "integrative literature"[tiab] OR "evidence-based review"[tiab] OR "evidence-based overview"[tiab] OR "evidence-based literature"[tiab] OR "evidence-based survey"[tiab] OR "literature search"[tiab] OR ((systemat*[ti] OR evidence-based[ti]) AND (review*[ti] OR literature[ti] OR overview[ti] OR survey[ti])) OR "data synthesis"[tiab] OR "evidence synthesis"[tiab] OR "data extraction"[tiab] OR "data source"[tiab] OR "data sources"[tiab] OR "study selection"[tiab] OR "methodological quality"[tiab] OR "methodologic quality"[tiab] OR cochrane database syst rev[ta] OR meta-analy*[tiab] OR metaanaly*[tiab] OR metanaly*[tiab] OR meta-analysis[pt] OR meta-synthesis[tiab] OR metasynthesis[tiab] OR meta-study[tiab] OR metastudy[tiab] OR metaethnograph*[tiab] OR meta-ethnograph*[tiab] OR Technology Assessment, Biomedical[mh] OR hta[tiab] OR health technol assess[ta] OR evid rep technol assess summ[ta] OR health technology assessment[tiab]) OR ((review*[ot] OR search*[ot] OR survey*[ot] OR handsearch*[ot] OR hand-search*[ot]) AND (databa*[ot] OR data-ba*[ot] OR bibliograph*[ot] OR electronic*[ot] OR medline*[ot] OR pubmed*[ot] OR embase*[ot] OR cochrane[ot] OR cinahl[ot] OR psycinfo[ot] OR psychinfo[ot] OR cinhal[ot] OR "web of science"[ot] OR "web of knowledge"[ot] OR ebsco[ot] OR ovid[ot] OR mrct[ot] OR metaregist*[ot] OR meta-regist*[ot] OR ((predetermined[ot] OR pre-determined[ot]) AND criteri*[ot]) OR apprais*[ot] OR inclusion criteri*[ot] OR exclusion criteri*[ot]) OR (review[pt] AND systemat*[ot]) OR "systematic review"[ot] OR "systematic literature"[ot] OR "integrative review"[ot] OR "integrative literature"[ot] OR "evidence-based review"[ot] OR "evidence-based overview"[ot] OR "evidence-based literature"[ot] OR "evidence-based survey"[ot] OR "literature search"[ot] OR ((systemat*[ti] OR evidence-based[ti]) AND (review*[ti] OR literature[ti] OR overview[ti] OR survey[ti])) OR "data synthesis"[ot] OR "evidence synthesis"[ot] OR "data extraction"[ot] OR "data source"[ot] OR "data sources"[ot] OR "study selection"[ot] OR "methodological quality"[ot] OR "methodologic quality"[ot] OR meta-analy*[ot] OR metaanaly*[ot] OR metanaly*[ot] OR meta-analysis[pt] OR meta-synthesis[ot] OR metasynthesis[ot] OR meta-study[ot] OR metastudy[ot] OR metaethnograph*[ot] OR meta-ethnograph*[ot] OR hta[ot] OR health technology assessment[ot])))) OR ((("Postoperative Care"[Majr] OR "Postoperative Complications"[Majr] OR "Postoperative Period"[Majr] OR "Recurrence"[Majr] OR "Patient Discharge"[Majr] OR "Cicatrix"[Majr] OR "Tissue Adhesions"[Majr] OR "Intestinal Obstruction"[Majr] OR "Infertility"[Majr] OR "Iatrogenic Disease"[Majr] OR postoperati*[ti] OR wound infecti*[ti] OR swi[ti] OR swis[ti] OR ssi[ti] OR ssis[ti] OR surgical site infecti*[ti] OR dehiscen*[ti] OR leak*[ti] OR discharg*[ti] OR fast track*[ti] OR fasttrack*[ti] OR scar[ti] OR scars[ti] OR scarring[ti] OR cicatri*[ti] OR stump*[ti] OR incision*[ti] OR adhesion*[ti] OR Intestinal Obstruction*[ti] OR ileus[ti] OR infertil*[ti] OR subfertil*[ti] OR iatrogen*[ti] OR postoperati*[ot] OR recurren*[ot] OR relaps*[ot] OR recrudescenc*[ot] OR wound infecti*[ot] OR swi[ot] OR swis[ot] OR ssi[ot] OR ssis[ot] OR surgical site infecti*[ot] OR dehiscen*[ot] OR leak*[ot] OR discharg*[ot] OR fast track*[ot] OR fasttrack*[ot] OR scar[ot] OR scars[ot] OR scarring[ot] OR cicatri*[ot] OR stump*[ot] OR incision*[ot] OR adhesion*[ot] OR Intestinal Obstruction*[ot] OR ileus[ot] OR infertil*[ot] OR subfertil*[ot] OR iatrogen*[ot]) AND ("Appendix"[Mesh] OR appendix[tiab] OR appendix[ot] OR "Appendicitis"[Mesh] OR "Appendectomy"[Mesh] OR appendicit*[tiab] OR appendicit*[ot] OR appendectom*[tiab] OR appendectom*[ot] OR appendicectom*[tiab] OR appendicectom*[ot]) NOT case reports[pt]) NOT (("Postoperative Care"[Mesh] OR "Postoperative Complications"[Mesh] OR "Postoperative Period"[Mesh] OR "Recurrence"[Mesh] OR "Patient Discharge"[Mesh] OR "Cicatrix"[Mesh] OR "Tissue Adhesions"[Mesh] OR "Intestinal Obstruction"[Mesh] OR "Infertility"[Mesh] OR "Iatrogenic Disease"[Mesh] OR postoperati*[tiab] OR wound infecti*[tiab] OR swi[tiab] OR swis[tiab] OR ssi[tiab] OR ssis[tiab] OR surgical site infecti*[tiab] OR dehiscen*[tiab] OR leak*[tiab] OR discharg*[tiab] OR fast track*[tiab] OR fasttrack*[tiab] OR scar[tiab] OR scars[tiab] OR scarring[tiab] OR cicatri*[tiab] OR stump*[tiab] OR incision*[tiab] OR adhesion*[tiab] OR Intestinal Obstruction*[tiab] OR ileus[tiab] OR infertil*[tiab] OR subfertil*[tiab] OR iatrogen*[tiab] OR postoperati*[ot] OR recurren*[ot] OR relaps*[ot] OR recrudescenc*[ot] OR wound infecti*[ot] OR swi[ot] OR swis[ot] OR ssi[ot] OR ssis[ot] OR surgical site infecti*[ot] OR dehiscen*[ot] OR leak*[ot] OR discharg*[ot] OR fast track*[ot] OR fasttrack*[ot] OR scar[ot] OR scars[ot] OR scarring[ot] OR cicatri*[ot] OR stump*[ot] OR incision*[ot] OR adhesion*[ot] OR Intestinal Obstruction*[ot] OR ileus[ot] OR infertil*[ot] OR subfertil*[ot] OR iatrogen*[ot]) AND ("Appendix"[Mesh] OR appendix[tiab] OR appendix[ot] OR "Appendicitis"[Mesh] OR "Appendectomy"[Mesh] OR appendicit*[tiab] OR appendicit*[ot] OR appendectom*[tiab] OR appendectom*[ot] OR appendicectom*[tiab] OR appendicectom*[ot]) AND (((review*[tiab] OR search*[tiab] OR survey*[tiab] OR handsearch*[tiab] OR hand-search*[tiab]) AND (databa*[tiab] OR data-ba*[tiab] OR bibliograph*[tiab] OR electronic*[tiab] OR medline*[tiab] OR pubmed*[tiab] OR embase*[tiab] OR Cochrane[tiab] OR cinahl[tiab] OR psycinfo[tiab] OR psychinfo[tiab] OR cinhal[tiab] OR "web of science"[tiab] OR "web of knowledge"[tiab] OR ebsco[tiab] OR ovid[tiab] OR mrct[tiab] OR metaregist*[tiab] OR meta-regist*[tiab] OR ((predetermined[tiab] OR pre-determined[tiab]) AND criteri*[tiab]) OR apprais*[tiab] OR inclusion criteri*[tiab] OR exclusion criteri*[tiab]) OR (review[pt] AND systemat*[tiab]) OR "systematic review"[tiab] OR "systematic literature"[tiab] OR "integrative review"[tiab] OR "integrative literature"[tiab] OR "evidence-based review"[tiab] OR "evidence-based overview"[tiab] OR "evidence-based literature"[tiab] OR "evidence-based survey"[tiab] OR "literature search"[tiab] OR ((systemat*[ti] OR evidence-based[ti]) AND (review*[ti] OR literature[ti] OR overview[ti] OR survey[ti])) OR "data synthesis"[tiab] OR "evidence synthesis"[tiab] OR "data extraction"[tiab] OR "data source"[tiab] OR "data sources"[tiab] OR "study selection"[tiab] OR "methodological quality"[tiab] OR "methodologic quality"[tiab] OR cochrane database syst rev[ta] OR meta-analy*[tiab] OR metaanaly*[tiab] OR metanaly*[tiab] OR meta-analysis[pt] OR meta-synthesis[tiab] OR metasynthesis[tiab] OR meta-study[tiab] OR metastudy[tiab] OR metaethnograph*[tiab] OR meta-ethnograph*[tiab] OR Technology Assessment, Biomedical[mh] OR hta[tiab] OR health technol assess[ta] OR evid rep technol assess summ[ta] OR health technology assessment[tiab]) OR ((review*[ot] OR search*[ot] OR survey*[ot] OR handsearch*[ot] OR hand-search*[ot]) AND (databa*[ot] OR data-ba*[ot] OR bibliograph*[ot] OR electronic*[ot] OR medline*[ot] OR pubmed*[ot] OR embase*[ot] OR cochrane[ot] OR cinahl[ot] OR psycinfo[ot] OR psychinfo[ot] OR cinhal[ot] OR "web of science"[ot] OR "web of knowledge"[ot] OR ebsco[ot] OR ovid[ot] OR mrct[ot] OR metaregist*[ot] OR meta-regist*[ot] OR ((predetermined[ot] OR pre-determined[ot]) AND criteri*[ot]) OR apprais*[ot] OR inclusion criteri*[ot] OR exclusion criteri*[ot]) OR (review[pt] AND systemat*[ot]) OR "systematic review"[ot] OR "systematic literature"[ot] OR "integrative review"[ot] OR "integrative literature"[ot] OR "evidence-based review"[ot] OR "evidence-based overview"[ot] OR "evidence-based literature"[ot] OR "evidence-based survey"[ot] OR "literature search"[ot] OR ((systemat*[ti] OR evidence-based[ti]) AND (review*[ti] OR literature[ti] OR overview[ti] OR survey[ti])) OR "data synthesis"[ot] OR "evidence synthesis"[ot] OR "data extraction"[ot] OR "data source"[ot] OR "data sources"[ot] OR "study selection"[ot] OR "methodological quality"[ot] OR "methodologic quality"[ot] OR meta-analy*[ot] OR metaanaly*[ot] OR metanaly*[ot] OR meta-analysis[pt] OR meta-synthesis[ot] OR metasynthesis[ot] OR meta-study[ot] OR metastudy[ot] OR metaethnograph*[ot] OR meta-ethnograph*[ot] OR hta[ot] OR health technology assessment[ot]))))) OR ((("Appendix"[Mesh] OR appendix[tiab] OR appendix[ot] OR "Appendicitis"[Mesh] OR "Appendectomy"[Mesh] OR appendicit*[tiab] OR appendicit*[ot] OR appendectom*[tiab] OR appendectom*[ot] OR appendicectom*[tiab] OR appendicectom*[ot]) AND (("Pathology"[Mesh] AND "pathology"[Subheading] OR patholog*[tiab] OR histopathol*[tiab] OR patholog*[ot] OR histopathol*[ot]) AND ("Incidence"[Mesh] OR incidenc*[tiab] OR incidenc*[ot]))) NOT case reports[pt])) OR ((("Appendix"[Mesh] OR appendix[tiab] OR appendix[ot] OR "Appendicitis"[Mesh] OR "Appendectomy"[Mesh] OR appendicit*[tiab] OR appendicit*[ot] OR appendectom*[tiab] OR appendectom*[ot] OR appendicectom*[tiab] OR appendicectom*[ot]) AND (("Learning Curve"[Mesh] OR learning curve*[tiab] OR skill*[tiab] OR expertise*[tiab]) OR "Professional Competence"[Mesh] OR competenc*[tiab]) AND ((therapy/narrow[filter]) OR (((review*[tiab] OR search*[tiab] OR survey*[tiab] OR handsearch*[tiab] OR hand-search*[tiab]) AND (databa*[tiab] OR data-ba*[tiab] OR bibliograph*[tiab] OR electronic*[tiab] OR medline*[tiab] OR pubmed*[tiab] OR embase*[tiab] OR Cochrane[tiab] OR cinahl[tiab] OR psycinfo[tiab] OR psychinfo[tiab] OR cinhal[tiab] OR "web of science"[tiab] OR "web of knowledge"[tiab] OR ebsco[tiab] OR ovid[tiab] OR mrct[tiab] OR metaregist*[tiab] OR meta-regist*[tiab] OR ((predetermined[tiab] OR pre-determined[tiab]) AND criteri*[tiab]) OR apprais*[tiab] OR inclusion criteri*[tiab] OR exclusion criteri*[tiab]) OR (review[pt] AND systemat*[tiab]) OR "systematic review"[tiab] OR "systematic literature"[tiab] OR "integrative review"[tiab] OR "integrative literature"[tiab] OR "evidence-based review"[tiab] OR "evidence-based overview"[tiab] OR "evidence-based literature"[tiab] OR "evidence-based survey"[tiab] OR "literature search"[tiab] OR ((systemat*[ti] OR evidence-based[ti]) AND (review*[ti] OR literature[ti] OR overview[ti] OR survey[ti])) OR "data synthesis"[tiab] OR "evidence synthesis"[tiab] OR "data extraction"[tiab] OR "data source"[tiab] OR "data sources"[tiab] OR "study selection"[tiab] OR "methodological quality"[tiab] OR "methodologic quality"[tiab] OR cochrane database syst rev[ta] OR meta-analy*[tiab] OR metaanaly*[tiab] OR metanaly*[tiab] OR meta-analysis[pt] OR meta-synthesis[tiab] OR metasynthesis[tiab] OR meta-study[tiab] OR metastudy[tiab] OR metaethnograph*[tiab] OR meta-ethnograph*[tiab] OR Technology Assessment, Biomedical[mh] OR hta[tiab] OR health technol assess[ta] OR evid rep technol assess summ[ta] OR health technology assessment[tiab]) OR ((review*[ot] OR search*[ot] OR survey*[ot] OR handsearch*[ot] OR hand-search*[ot]) AND (databa*[ot] OR data-ba*[ot] OR bibliograph*[ot] OR electronic*[ot] OR medline*[ot] OR pubmed*[ot] OR embase*[ot] OR cochrane[ot] OR cinahl[ot] OR psycinfo[ot] OR psychinfo[ot] OR cinhal[ot] OR "web of science"[ot] OR "web of knowledge"[ot] OR ebsco[ot] OR ovid[ot] OR mrct[ot] OR metaregist*[ot] OR meta-regist*[ot] OR ((predetermined[ot] OR pre-determined[ot]) AND criteri*[ot]) OR apprais*[ot] OR inclusion criteri*[ot] OR exclusion criteri*[ot]) OR (review[pt] AND systemat*[ot]) OR "systematic review"[ot] OR "systematic literature"[ot] OR "integrative review"[ot] OR "integrative literature"[ot] OR "evidence-based review"[ot] OR "evidence-based overview"[ot] OR "evidence-based literature"[ot] OR "evidence-based survey"[ot] OR "literature search"[ot] OR ((systemat*[ti] OR evidence-based[ti]) AND (review*[ti] OR literature[ti] OR overview[ti] OR survey[ti])) OR "data synthesis"[ot] OR "evidence synthesis"[ot] OR "data extraction"[ot] OR "data source"[ot] OR "data sources"[ot] OR "study selection"[ot] OR "methodological quality"[ot] OR "methodologic quality"[ot] OR meta-analy*[ot] OR metaanaly*[ot] OR metanaly*[ot] OR meta-analysis[pt] OR meta-synthesis[ot] OR metasynthesis[ot] OR meta-study[ot] OR metastudy[ot] OR metaethnograph*[ot] OR meta-ethnograph*[ot] OR hta[ot] OR health technology assessment[ot])))) OR (("Appendix"[Mesh] OR appendix[tiab] OR appendix[ot] OR "Appendicitis"[Mesh] OR "Appendectomy"[Mesh] OR appendicit*[tiab] OR appendicit*[ot] OR appendectom*[tiab] OR appendectom*[ot] OR appendicectom*[tiab] OR appendicectom*[ot]) AND (("Learning Curve"[Majr] OR learning curve*[ti] OR skill*[ti] OR expertise*[ti]) OR "Professional Competence"[Majr] OR competenc*[ti])) NOT case reports[pt])) OR (((((timing*[ti] OR delay*[ti] OR time frame*[ti] OR period*[ti])) OR ("Time Factors"[Majr])) AND ("Appendix"[Mesh] OR appendix[tiab] OR appendix[ot] OR "Appendicitis"[Mesh] OR "Appendectomy"[Mesh] OR appendicit*[tiab] OR appendicit*[ot] OR appendectom*[tiab] OR appendectom*[ot] OR appendicectom*[tiab] OR appendicectom*[ot])) OR ((("Appendix"[Mesh] OR appendix[tiab] OR appendix[ot] OR "Appendicitis"[Mesh] OR "Appendectomy"[Mesh] OR appendicit*[tiab] OR appendicit*[ot] OR appendectom*[tiab] OR appendectom*[ot] OR appendicectom*[tiab] OR appendicectom*[ot]) AND (timing[tiab] OR delay[tiab] OR time frame*[tiab])) AND ((((review*[tiab] OR search*[tiab] OR survey*[tiab] OR handsearch*[tiab] OR hand-search*[tiab]) AND (databa*[tiab] OR data-ba*[tiab] OR bibliograph*[tiab] OR electronic*[tiab] OR medline*[tiab] OR pubmed*[tiab] OR embase*[tiab] OR Cochrane[tiab] OR cinahl[tiab] OR psycinfo[tiab] OR psychinfo[tiab] OR cinhal[tiab] OR "web of science"[tiab] OR "web of knowledge"[tiab] OR ebsco[tiab] OR ovid[tiab] OR mrct[tiab] OR metaregist*[tiab] OR meta-regist*[tiab] OR ((predetermined[tiab] OR pre-determined[tiab]) AND criteri*[tiab]) OR apprais*[tiab] OR inclusion criteri*[tiab] OR exclusion criteri*[tiab]) OR (review[pt] AND systemat*[tiab]) OR "systematic review"[tiab] OR "systematic literature"[tiab] OR "integrative review"[tiab] OR "integrative literature"[tiab] OR "evidence-based review"[tiab] OR "evidence-based overview"[tiab] OR "evidence-based literature"[tiab] OR "evidence-based survey"[tiab] OR "literature search"[tiab] OR ((systemat*[ti] OR evidence-based[ti]) AND (review*[ti] OR literature[ti] OR overview[ti] OR survey[ti])) OR "data synthesis"[tiab] OR "evidence synthesis"[tiab] OR "data extraction"[tiab] OR "data source"[tiab] OR "data sources"[tiab] OR "study selection"[tiab] OR "methodological quality"[tiab] OR "methodologic quality"[tiab] OR cochrane database syst rev[ta] OR meta-analy*[tiab] OR metaanaly*[tiab] OR metanaly*[tiab] OR meta-analysis[pt] OR meta-synthesis[tiab] OR metasynthesis[tiab] OR meta-study[tiab] OR metastudy[tiab] OR metaethnograph*[tiab] OR meta-ethnograph*[tiab] OR Technology Assessment, Biomedical[mh] OR hta[tiab] OR health technol assess[ta] OR evid rep technol assess summ[ta] OR health technology assessment[tiab]) OR ((review*[ot] OR search*[ot] OR survey*[ot] OR handsearch*[ot] OR hand-search*[ot]) AND (databa*[ot] OR data-ba*[ot] OR bibliograph*[ot] OR electronic*[ot] OR medline*[ot] OR pubmed*[ot] OR embase*[ot] OR cochrane[ot] OR cinahl[ot] OR psycinfo[ot] OR psychinfo[ot] OR cinhal[ot] OR "web of science"[ot] OR "web of knowledge"[ot] OR ebsco[ot] OR ovid[ot] OR mrct[ot] OR metaregist*[ot] OR meta-regist*[ot] OR ((predetermined[ot] OR pre-determined[ot]) AND criteri*[ot]) OR apprais*[ot] OR inclusion criteri*[ot] OR exclusion criteri*[ot]) OR (review[pt] AND systemat*[ot]) OR "systematic review"[ot] OR "systematic literature"[ot] OR "integrative review"[ot] OR "integrative literature"[ot] OR "evidence-based review"[ot] OR "evidence-based overview"[ot] OR "evidence-based literature"[ot] OR "evidence-based survey"[ot] OR "literature search"[ot] OR ((systemat*[ti] OR evidence-based[ti]) AND (review*[ti] OR literature[ti] OR overview[ti] OR survey[ti])) OR "data synthesis"[ot] OR "evidence synthesis"[ot] OR "data extraction"[ot] OR "data source"[ot] OR "data sources"[ot] OR "study selection"[ot] OR "methodological quality"[ot] OR "methodologic quality"[ot] OR meta-analy*[ot] OR metaanaly*[ot] OR metanaly*[ot] OR meta-analysis[pt] OR meta-synthesis[ot] OR metasynthesis[ot] OR meta-study[ot] OR metastudy[ot] OR metaethnograph*[ot] OR meta-ethnograph*[ot] OR hta[ot] OR health technology assessment[ot])) OR (therapy/narrow[filter]))) NOT case reports[pt])) OR ((("Appendix"[Mesh] OR appendix[tiab] OR appendix[ot] OR "Appendicitis"[Mesh] OR "Appendectomy"[Mesh] OR appendicit*[tiab] OR appendicit*[ot] OR appendectom*[tiab] OR appendectom*[ot] OR appendicectom*[tiab] OR appendicectom*[ot]) AND ("Antibiotic Prophylaxis"[Mesh] OR ((antibiotic*[tiab] OR antibacteri*[tiab]) AND (prophylax*[tiab] OR premedicati*[tiab])) OR "Intubation, Gastrointestinal"[Mesh] OR ((Nasogastric[tiab] OR gastric[tiab]) AND (intubation*[tiab] OR tube[tiab] OR tubes[tiab])) OR "Urinary Catheters"[Mesh] OR "Urinary Catheterization"[Mesh] OR (urin*[tiab] AND catheter*[tiab]) OR "Patient Positioning"[Mesh] OR ((patient*[tiab] OR table*[tiab] OR "Operating Tables"[Mesh]) AND position*[tiab]) OR "Pneumoperitoneum, Artificial"[Mesh] OR pneumoperiton*[tiab] OR veress*[tiab] OR trocar*[tiab] OR open introduc*[tiab]) AND ((therapy/narrow[filter]) OR (((review*[tiab] OR search*[tiab] OR survey*[tiab] OR handsearch*[tiab] OR hand-search*[tiab]) AND (databa*[tiab] OR data-ba*[tiab] OR bibliograph*[tiab] OR electronic*[tiab] OR medline*[tiab] OR pubmed*[tiab] OR embase*[tiab] OR Cochrane[tiab] OR cinahl[tiab] OR psycinfo[tiab] OR psychinfo[tiab] OR cinhal[tiab] OR "web of science"[tiab] OR "web of knowledge"[tiab] OR ebsco[tiab] OR ovid[tiab] OR mrct[tiab] OR metaregist*[tiab] OR meta-regist*[tiab] OR ((predetermined[tiab] OR pre-determined[tiab]) AND criteri*[tiab]) OR apprais*[tiab] OR inclusion criteri*[tiab] OR exclusion criteri*[tiab]) OR (review[pt] AND systemat*[tiab]) OR "systematic review"[tiab] OR "systematic literature"[tiab] OR "integrative review"[tiab] OR "integrative literature"[tiab] OR "evidence-based review"[tiab] OR "evidence-based overview"[tiab] OR "evidence-based literature"[tiab] OR "evidence-based survey"[tiab] OR "literature search"[tiab] OR ((systemat*[ti] OR evidence-based[ti]) AND (review*[ti] OR literature[ti] OR overview[ti] OR survey[ti])) OR "data synthesis"[tiab] OR "evidence synthesis"[tiab] OR "data extraction"[tiab] OR "data source"[tiab] OR "data sources"[tiab] OR "study selection"[tiab] OR "methodological quality"[tiab] OR "methodologic quality"[tiab] OR cochrane database syst rev[ta] OR meta-analy*[tiab] OR metaanaly*[tiab] OR metanaly*[tiab] OR meta-analysis[pt] OR meta-synthesis[tiab] OR metasynthesis[tiab] OR meta-study[tiab] OR metastudy[tiab] OR metaethnograph*[tiab] OR meta-ethnograph*[tiab] OR Technology Assessment, Biomedical[mh] OR hta[tiab] OR health technol assess[ta] OR evid rep technol assess summ[ta] OR health technology assessment[tiab]) OR ((review*[ot] OR search*[ot] OR survey*[ot] OR handsearch*[ot] OR hand-search*[ot]) AND (databa*[ot] OR data-ba*[ot] OR bibliograph*[ot] OR electronic*[ot] OR medline*[ot] OR pubmed*[ot] OR embase*[ot] OR cochrane[ot] OR cinahl[ot] OR psycinfo[ot] OR psychinfo[ot] OR cinhal[ot] OR "web of science"[ot] OR "web of knowledge"[ot] OR ebsco[ot] OR ovid[ot] OR mrct[ot] OR metaregist*[ot] OR meta-regist*[ot] OR ((predetermined[ot] OR pre-determined[ot]) AND criteri*[ot]) OR apprais*[ot] OR inclusion criteri*[ot] OR exclusion criteri*[ot]) OR (review[pt] AND systemat*[ot]) OR "systematic review"[ot] OR "systematic literature"[ot] OR "integrative review"[ot] OR "integrative literature"[ot] OR "evidence-based review"[ot] OR "evidence-based overview"[ot] OR "evidence-based literature"[ot] OR "evidence-based survey"[ot] OR "literature search"[ot] OR ((systemat*[ti] OR evidence-based[ti]) AND (review*[ti] OR literature[ti] OR overview[ti] OR survey[ti])) OR "data synthesis"[ot] OR "evidence synthesis"[ot] OR "data extraction"[ot] OR "data source"[ot] OR "data sources"[ot] OR "study selection"[ot] OR "methodological quality"[ot] OR "methodologic quality"[ot] OR meta-analy*[ot] OR metaanaly*[ot] OR metanaly*[ot] OR meta-analysis[pt] OR meta-synthesis[ot] OR metasynthesis[ot] OR meta-study[ot] OR metastudy[ot] OR metaethnograph*[ot] OR meta-ethnograph*[ot] OR hta[ot] OR health technology assessment[ot])))) OR (("Appendix"[Mesh] OR appendix[tiab] OR appendix[ot] OR "Appendicitis"[Mesh] OR "Appendectomy"[Mesh] OR appendicit*[tiab] OR appendicit*[ot] OR appendectom*[tiab] OR appendectom*[ot] OR appendicectom*[tiab] OR appendicectom*[ot]) AND ("Antibiotic Prophylaxis"[Majr] OR ((antibiotic*[ti] OR antibacteri*[ti]) AND (prophylax*[ti] OR premedicati*[ti])) OR "Intubation, Gastrointestinal"[Majr] OR ((Nasogastric[ti] OR gastric[ti]) AND (intubation*[ti] OR tube[ti] OR tubes[ti])) OR "Urinary Catheters"[Majr] OR "Urinary Catheterization"[Majr] OR (urin*[ti] AND catheter*[ti]) OR "Patient Positioning"[Majr] OR ((patient*[ti] OR table*[ti] OR "Operating Tables"[Majr]) AND position*[ti]) OR "Pneumoperitoneum, Artificial"[Majr] OR pneumoperiton*[ti] OR veress*[ti] OR trocar*[ti] OR open introduc*[ti])) NOT case reports[pt])) OR (((("Appendix"[Mesh] OR appendix[tiab] OR appendix[ot] OR "Appendicitis"[Mesh] OR "Appendectomy"[Mesh] OR appendicit*[tiab] OR appendicit*[ot] OR appendectom*[tiab] OR appendectom*[ot] OR appendicectom*[tiab] OR appendicectom*[ot]) AND (appendix sana[tiab] OR "Surgical Instruments"[Mesh] OR clip*[tiab] OR stapl*[tiab] OR endoclip*[tiab] OR endostapl*[tiab] OR loop[tiab] OR loops[tiab] OR endoloop*[tiab] OR skeletiz*[tiab] OR skeletis*[tiab] OR "Diathermy"[Mesh] OR diatherm*[tiab] OR "Sutures"[Mesh] OR sutur*[tiab] OR endobag*[tiab] OR glove*[tiab] OR "Gloves, Surgical"[Mesh] OR trocar*[tiab] OR extracti*[tiab] OR ((intraabdom*[tiab] OR abdom*[tiab]) AND fluid*[tiab] OR purulen*[tiab]) OR "Suppuration"[Mesh] OR pus[tiab] OR suppurati*[tiab] OR abscess*[tiab] OR "Conversion to Open Surgery"[Mesh] OR conver*[tiab] OR culture*[tiab] OR swab*[tiab]) AND ((((review*[tiab] OR search*[tiab] OR survey*[tiab] OR handsearch*[tiab] OR hand-search*[tiab]) AND (databa*[tiab] OR data-ba*[tiab] OR bibliograph*[tiab] OR electronic*[tiab] OR medline*[tiab] OR pubmed*[tiab] OR embase*[tiab] OR Cochrane[tiab] OR cinahl[tiab] OR psycinfo[tiab] OR psychinfo[tiab] OR cinhal[tiab] OR "web of science"[tiab] OR "web of knowledge"[tiab] OR ebsco[tiab] OR ovid[tiab] OR mrct[tiab] OR metaregist*[tiab] OR meta-regist*[tiab] OR ((predetermined[tiab] OR pre-determined[tiab]) AND criteri*[tiab]) OR apprais*[tiab] OR inclusion criteri*[tiab] OR exclusion criteri*[tiab]) OR (review[pt] AND systemat*[tiab]) OR "systematic review"[tiab] OR "systematic literature"[tiab] OR "integrative review"[tiab] OR "integrative literature"[tiab] OR "evidence-based review"[tiab] OR "evidence-based overview"[tiab] OR "evidence-based literature"[tiab] OR "evidence-based survey"[tiab] OR "literature search"[tiab] OR ((systemat*[ti] OR evidence-based[ti]) AND (review*[ti] OR literature[ti] OR overview[ti] OR survey[ti])) OR "data synthesis"[tiab] OR "evidence synthesis"[tiab] OR "data extraction"[tiab] OR "data source"[tiab] OR "data sources"[tiab] OR "study selection"[tiab] OR "methodological quality"[tiab] OR "methodologic quality"[tiab] OR cochrane database syst rev[ta] OR meta-analy*[tiab] OR metaanaly*[tiab] OR metanaly*[tiab] OR meta-analysis[pt] OR meta-synthesis[tiab] OR metasynthesis[tiab] OR meta-study[tiab] OR metastudy[tiab] OR metaethnograph*[tiab] OR meta-ethnograph*[tiab] OR Technology Assessment, Biomedical[mh] OR hta[tiab] OR health technol assess[ta] OR evid rep technol assess summ[ta] OR health technology assessment[tiab]) OR ((review*[ot] OR search*[ot] OR survey*[ot] OR handsearch*[ot] OR hand-search*[ot]) AND (databa*[ot] OR data-ba*[ot] OR bibliograph*[ot] OR electronic*[ot] OR medline*[ot] OR pubmed*[ot] OR embase*[ot] OR cochrane[ot] OR cinahl[ot] OR psycinfo[ot] OR psychinfo[ot] OR cinhal[ot] OR "web of science"[ot] OR "web of knowledge"[ot] OR ebsco[ot] OR ovid[ot] OR mrct[ot] OR metaregist*[ot] OR meta-regist*[ot] OR ((predetermined[ot] OR pre-determined[ot]) AND criteri*[ot]) OR apprais*[ot] OR inclusion criteri*[ot] OR exclusion criteri*[ot]) OR (review[pt] AND systemat*[ot]) OR "systematic review"[ot] OR "systematic literature"[ot] OR "integrative review"[ot] OR "integrative literature"[ot] OR "evidence-based review"[ot] OR "evidence-based overview"[ot] OR "evidence-based literature"[ot] OR "evidence-based survey"[ot] OR "literature search"[ot] OR ((systemat*[ti] OR evidence-based[ti]) AND (review*[ti] OR literature[ti] OR overview[ti] OR survey[ti])) OR "data synthesis"[ot] OR "evidence synthesis"[ot] OR "data extraction"[ot] OR "data source"[ot] OR "data sources"[ot] OR "study selection"[ot] OR "methodological quality"[ot] OR "methodologic quality"[ot] OR meta-analy*[ot] OR metaanaly*[ot] OR metanaly*[ot] OR meta-analysis[pt] OR meta-synthesis[ot] OR metasynthesis[ot] OR meta-study[ot] OR metastudy[ot] OR metaethnograph*[ot] OR meta-ethnograph*[ot] OR hta[ot] OR health technology assessment[ot])) OR (therapy/narrow[filter])))) OR (("Appendix"[Mesh] OR appendix[tiab] OR appendix[ot] OR "Appendicitis"[Mesh] OR "Appendectomy"[Mesh] OR appendicit*[tiab] OR appendicit*[ot] OR appendectom*[tiab] OR appendectom*[ot] OR appendicectom*[tiab] OR appendicectom*[ot]) AND (appendix sana[ti] OR "Surgical Instruments"[majr] OR clip*[ti] OR stapl*[ti] OR endoclip*[ti] OR endostapl*[ti] OR loop[ti] OR loops[ti] OR endoloop*[ti] OR skeletiz*[ti] OR skeletis*[ti] OR "Diathermy"[majr] OR diatherm*[ti] OR "Sutures"[majr] OR sutur*[ti] OR endobag*[ti] OR glove*[ti] OR "Gloves, Surgical"[majr] OR trocar*[ti] OR extracti*[ti] OR ((intraabdom*[ti] OR abdom*[ti]) AND fluid*[ti] OR purulen*[ti]) OR "Suppuration"[majr] OR pus[ti] OR suppurati*[ti] OR abscess*[ti] OR "Conversion to Open Surgery"[majr] OR conver*[ti] OR culture*[ti] OR swab*[ti])) NOT case reports[pt])) OR (((("Appendix"[Mesh] OR appendix[tiab] OR appendix[ot] OR "Appendicitis"[Mesh] OR "Appendectomy"[Mesh] OR appendicit*[tiab] OR appendicit*[ot] OR appendectom*[tiab] OR appendectom*[ot] OR appendicectom*[tiab] OR appendicectom*[ot]) AND ("Microbiology"[Mesh] OR "Bacteria"[Mesh] OR bacteriolog*[tiab] OR bacteria*[tiab] OR microbiolog*[tiab] OR bacteriolog*[ot] OR bacteria*[ot] OR microbiolog*[ot])) NOT case reports[pt]))** | 8147 |
