# Supplementary material for: Diagnosis and management of acute appendicitis. EAES consensus development conference 2015
Source: Surg Endosc. 2016 Sep 22;30(11):4668–90. doi: 10.1007/s00464-016-5245-7 (PMC5082605; doi:10.1007/s00464-016-5245-7)
Supplement: Supplementary file 3 — Supplementary material 3 (DOCX 87 kb) [file 464_2016_5245_MOESM3_ESM.docx]

Appendix #3

Number of articles actually used per topic

| Main topic |  | Number of articles |
| --- | --- | --- |
| Pre-operative care |  |  |
|  | Diagnostic work-up | 117 |
|  | Treatment indications | 124 |
| Operative care |  |  |
| *General aspects* | Timing | 26 |
| *Before surgery* | Antibiotic prophylaxis | 37 |
|  | Nasogastric tube, urinary catheter and positioning | 19 |
|  | Technique | 34 |
| *Intra-operative* | Intra-operative | 70 |
|  | Intra-operative unexpected findings | 13 |
| After care |  |  |
|  | Postoperative antibiotics | 24 |
|  | Postoperative complications | 31 |
|  | Postoperative care | 22 |
|  | Pathology | 58 |
|  |  |  |
| Total |  | **575** |
